# Structure of the *Escherichia coli* ProQ RNA-binding protein

**DOI:** 10.1261/rna.060343.116

**Published:** 2017-05

**Authors:** Grecia M. Gonzalez, Steven W. Hardwick, Sarah L. Maslen, J. Mark Skehel, Erik Holmqvist, Jörg Vogel, Alex Bateman, Ben F. Luisi, R. William Broadhurst

**Affiliations:** 1 Department of Biochemistry, University of Cambridge, Cambridge CB2 1GA, United Kingdom; 2 MRC Laboratory of Molecular Biology, Cambridge CB2 0QH, United Kingdom; 3 Department of Cell and Molecular Biology, Biomedical Center, Uppsala University, 75124 Uppsala, Sweden; 4 RNA Biology Group, Institute of Molecular Infection Biology, University of Würzburg, D-97080 Wurzburg, Germany; 5 Helmholtz Institute for RNA-based Infection Research (HIRI), University of Würzburg, D-97080 Wurzburg, Germany; 6 European Molecular Biology Laboratory, European Bioinformatics Institute (EMBL-EBI), Wellcome Genome Campus, Hinxton, Cambridge CB10 1SD, United Kingdom

**Keywords:** protein–RNA interactions, regulatory RNA, riboregulation, FinO, ProQ, RNA chaperone

## Abstract

The protein ProQ has recently been identified as a global small noncoding RNA-binding protein in *Salmonella*, and a similar role is anticipated for its numerous homologs in divergent bacterial species. We report the solution structure of *Escherichia coli* ProQ, revealing an N-terminal FinO-like domain, a C-terminal domain that unexpectedly has a Tudor domain fold commonly found in eukaryotes, and an elongated bridging intradomain linker that is flexible but nonetheless incompressible. Structure-based sequence analysis suggests that the Tudor domain was acquired through horizontal gene transfer and gene fusion to the ancestral FinO-like domain. Through a combination of biochemical and biophysical approaches, we have mapped putative RNA-binding surfaces on all three domains of ProQ and modeled the protein's conformation in the *apo* and RNA-bound forms. Taken together, these data suggest how the FinO, Tudor, and linker domains of ProQ cooperate to recognize complex RNA structures and serve to promote RNA-mediated regulation.

## INTRODUCTION

In all domains of life, regulatory RNAs are versatile modulators of gene expression, and their actions are facilitated by protein chaperones with which they cooperate to achieve specificity and rapid response (Wagner and Romby 2015). In the last decade, small RNAs (sRNAs) have emerged as an important class of gene regulators in bacteria, contributing to intricate post-transcriptional networks and providing controlled responses to diverse types of stress, metabolic changes, and extracellular signals (Gottesman and Storz 2011). Ranging in size from 50–250 nucleotides (nt), sRNAs typically act by imperfectly base-pairing with their mRNA targets using a cognate seed region. This recognition can result in different downstream effects, depending on context. In many cases, association of an sRNA near a target transcript's ribosome binding site may either mask or expose an RNA element to alter the translation efficiency of the mRNA. In other cases, the sRNA:mRNA interaction can target the mRNA for rapid degradation, often by the multienzyme RNA degradosome assembly (Fei et al. 2015; Wagner and Romby 2015). sRNAs can also operate on actively transcribed genes by modulating the process of transcription termination (Sedlyarova et al. 2016).

To date, a small number of proteins have been identified that specifically bind to sRNA molecules in numerous bacterial species, including Hfq, CsrA, and more recently FinO-like proteins (Romeo et al. 2013; Wagner and Romby 2015; Attaiech et al. 2016; Smirnov et al. 2016, 2017). The protein ProQ, a FinO family member, was originally identified as an osmoregulatory factor required for optimal expression of the proline channel protein ProP (Kunte et al. 1999; Chaulk et al. 2011; Kerr et al. 2014). However, ProQ has now emerged to function more broadly as a global sRNA binding protein in *Salmonella*, and a similar role is predicted for proteins containing ProQ/FinO domains found in divergent bacterial species (Smirnov et al. 2016). In *Legionella pneumophila*, a ProQ/FinO domain protein has been shown to be required for regulation of an sRNA controlling natural competence (Attaiech et al. 2016).

The N-terminal domain of *Escherichia coli* ProQ, spanning residues 1-121, is composed of a ProQ/FinO domain (PFAM04352), and shares 35% sequence identity with its paralog FinO (Attaiech et al. 2017) and is predicted to have significant structural similarities (Glover et al. 2015). Crystallographic studies of *Neisseria meningitidis* ProQ/FinO domain protein (Chaulk et al. 2010) corroborate the anticipated close structural relationship of this domain with FinO (Ghetu et al. 2000). Given their predicted structural similarity, the ProQ/FinO domain of FinO and the ProQ N-terminal domain are likely to share similar modes of RNA interaction. FinO controls the function of the antisense RNA FinP, which represses the conjugative transfer of IncF plasmids by base-pairing to the conserved stem–loops of the 5′ untranslated region of the *traJ* transcript encoding a transcriptional activator (Van Biesen and Frost 1994; Arthur et al. 2003). ProQ has also been shown to have a propensity to interact with structured RNA, including sRNAs and mRNAs (Smirnov et al. 2016).

**FIGURE 1.**
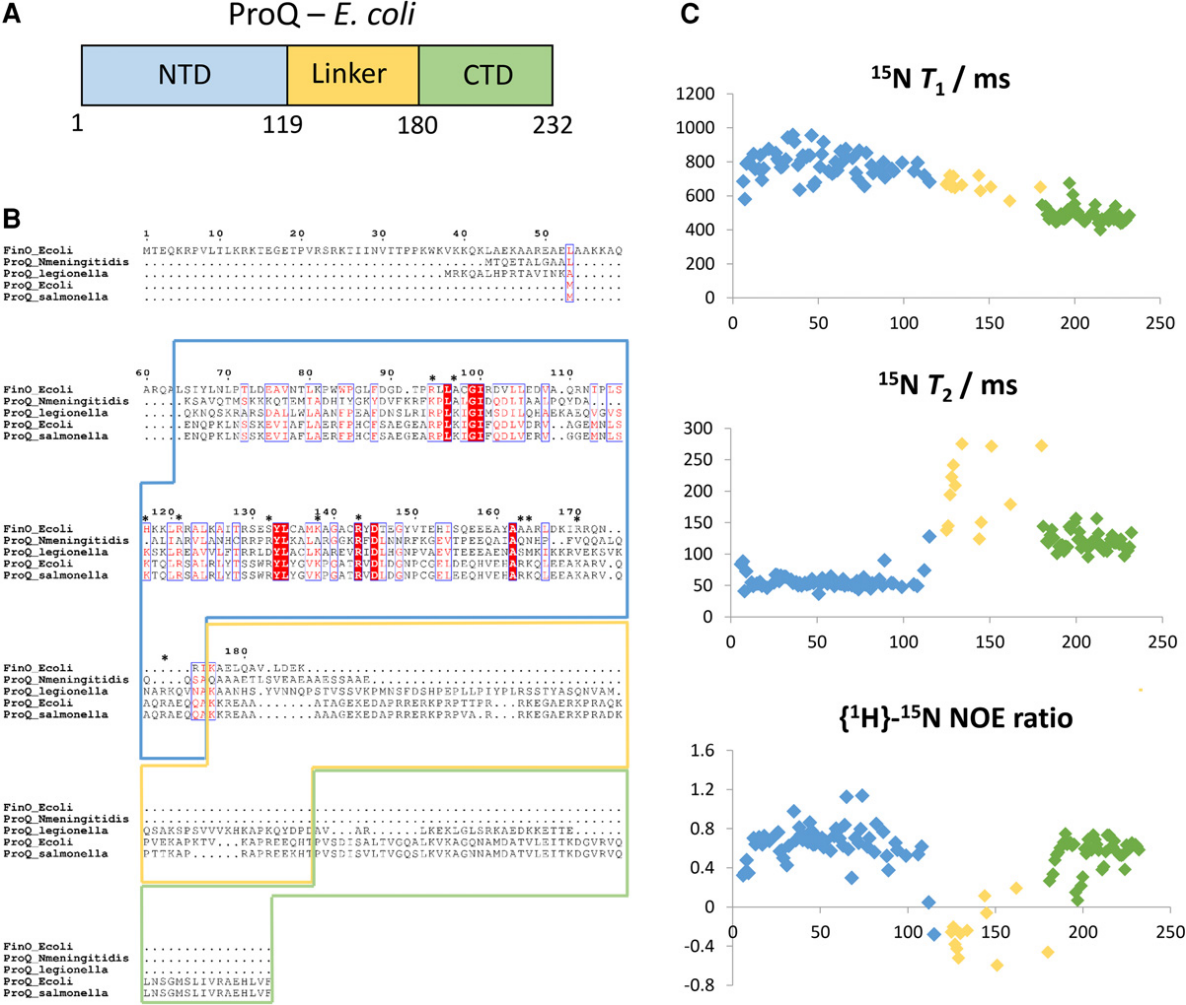
Domain organization and NMR spectroscopy of ProQ. (*A*) Linear domain representation of ProQ, with the ProQ/FinO N-terminal domain (NTD, blue), the disordered linker region (yellow), and the C-terminal domain (CTD, green). (*B*) Sequence alignment of ProQ proteins from *Salmonella enterica*, *E. coli*, *Legionella pneumophila*, *Neisseria meningitdis*, and FinO from *E. coli*. Sequence regions corresponding to the NTD, linker, and CTD are highlighted by boxes colored as in *A*. Electro-positive residues predicted to be involved in RNA binding (from Fig. 2) are indicated with asterisks. (*C*) ^15^N relaxation data for full length *apo* ProQ. Per residue plots for *T*_1_, *T*_2_, and the {^1^H}-^15^N heteronuclear NOE ratio are shown in the *top*, *middle*, and *bottom* panels, respectively. Individual data points are colored blue, yellow, or green to signify whether they correspond to residues from the NTD, linker, or CTD, respectively.

Limited proteolysis experiments have previously indicated that ProQ has a multidomain architecture, with globular N- and C-terminal domains (residues 1–130 and 180–232, respectively) separated by a disordered linker region (Fig. 1A; Smith et al. 2007). Presently, the structure of the C-terminal domain of ProQ is unknown, and it is not clear if it is able to bind RNA through a cooperative interplay with the N-terminal domain.

While the structural data for the *N. meningitidis* ProQ/FinO domain protein has been helpful to visualize the fold of the N-terminal domain, there are no structural data presently available to illuminate the function of the full length ProQ proteins of *E. coli* and *Salmonella*, organisms for which ProQ is now established as a global sRNA binding protein (Smirnov et al. 2016). To address how the complete ProQ is folded and recognizes RNA, we have structurally characterized the *E. coli* protein, which is nearly identical (92.2%) to the *Salmonella* ProQ. We present NMR solution structure ensembles for the isolated N- and C-terminal domains of ProQ, together with SAXS (small-angle X-ray solution scattering) models of the full length protein in complex with RNA. Using hydrogen–deuterium exchange (HDX) mass spectrometry, we map the binding surfaces on ProQ for two known targets: a 3′ untranslated region (3′ UTR) and a small regulatory RNA. While the results confirm that the NTD of *E. coli* ProQ has a conserved ProQ/FinO domain, they surprisingly reveal that the C-terminal domain is structurally related to the Tudor-like domains commonly found in eukaryotic chromatin regulators. The linker between these domains is highly flexible but extended, so that the molecule has an elongated shape that does not compact even in the presence of cognate RNA. We discuss models for how ProQ recognizes RNA and the molecular origins of the ProQ family.

## RESULTS

### NMR analysis of full length ProQ

Previous studies have suggested that ProQ is a multidomain protein with a well-conserved globular N-terminal FinO-like domain, a more variable globular C-terminal domain, and a disordered connector region (Fig. 1A,B; Smith et al. 2007). In solution, ProQ is monomeric, as indicated by the experimentally measured molecular mass of 25,490 g mol^–1^ determined by SEC-MALS (size exclusion chromatography combined with multiangle laser light scattering) and analytical ultracentrifugation (Supplemental Fig. S1), and in accord with reported results from MALDI-TOF mass spectrometry (Smith et al. 2004). Given the relatively small size of the *apo* species in solution (25 kDa), NMR spectroscopy was used as the method of choice for structural characterization of ProQ.

Using a uniformly ^13^C/^15^N-labeled sample of full length ProQ, nearly complete backbone and side-chain assignments were obtained for the C-terminal domain spanning residues 180–232, and partial sequence assignments were deduced for the N-terminal FinO-like domain (residues 1–119) and the interdomain linker region. In the [^1^H,^15^N]-HSQC (heteronuclear single quantum coherence) spectrum (Supplemental Fig. S2), the chemical shifts of backbone amide signals were well dispersed for the N- and C-terminal domains, consistent with the formation of tertiary structure. In contrast, resonances from the interdomain linker were poorly dispersed. Ratios of the ^15^N relaxation parameters *T*_1_ and *T*_2_ (Fig. 1C, top and middle panels) provide estimated overall rotational correlation time values of 9.6 and 6.7 nsec for the N- and C-terminal domains, respectively. The semi-independent rotational diffusion of the two domains indicates that they do not mutually interact in the RNA-free *apo* state. The interdomain linker region has low or negative values for the steady-state {^1^H}-^15^N NOE (nuclear Overhauser effect) ratio, which are characteristic of backbone amide sites that are highly flexible and possess extensive local motions on the sub-nanosecond timescale (Fig. 1C, bottom panel).

### The NMR structure of the N-terminal domain of ProQ

**FIGURE 2.**
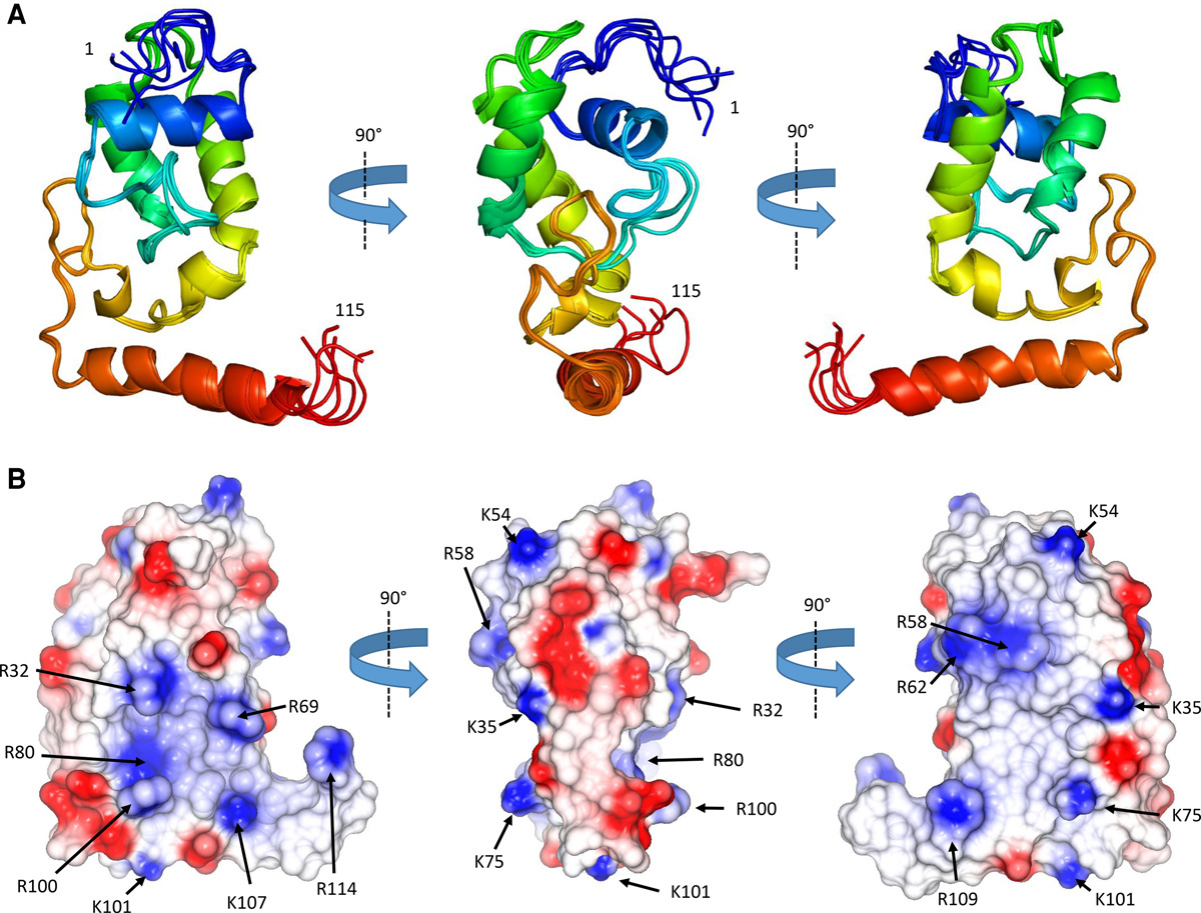
The NMR structure of the N-terminal domain of ProQ. (*A*) Ensemble of the top five solutions shown as cartoon representation in three orientations, and colored as rainbow from blue to red. (*B*) Electrostatic surface representation of the ProQ NTD, three views as in Figure 2A (generated in Coot) (Emsley et al. 2010). Electro-negative patches are colored red and electro-positive patches are colored blue. Residues forming putative RNA-binding surfaces are indicated with arrows.

The N-terminal domain (NTD) was characterized further using a uniformly ^13^C/^15^N-labeled fragment of ProQ spanning residues 1–119, preceded by a 14-residue sequence comprising a His_6_-tag and a short flexible linker. The [^1^H, ^15^N]-HSQC spectrum for the NTD alone (Supplemental Fig. S3) overlays closely with the corresponding signals from the [^1^H, ^15^N]-HSQC spectrum of full length ProQ, indicating that in the context of the full length protein residues of the NTD are not involved in additional contacts with residues from the linker region or C-terminal domain. The average HN chemical shift difference between residues in the NTD and full length ProQ samples are plotted in Supplemental Figure S4. The spectra differ close to histidine residues (23H, 95H, and 98H); however, this is common for samples prepared at slightly different pH values (pH 7.0 for full length ProQ and 6.5 for NTD). A total of 3142 NOE-derived distance restraints, of which 2388 were unambiguously assigned, were used in the structure calculations. After water refinement, the final ensemble comprising the 20 lowest energy structures has a backbone coordinate root mean square deviation (RMSD) of 0.58 Å over residues 5–111 (Table 1; Fig. 2A).

**TABLE 1.**
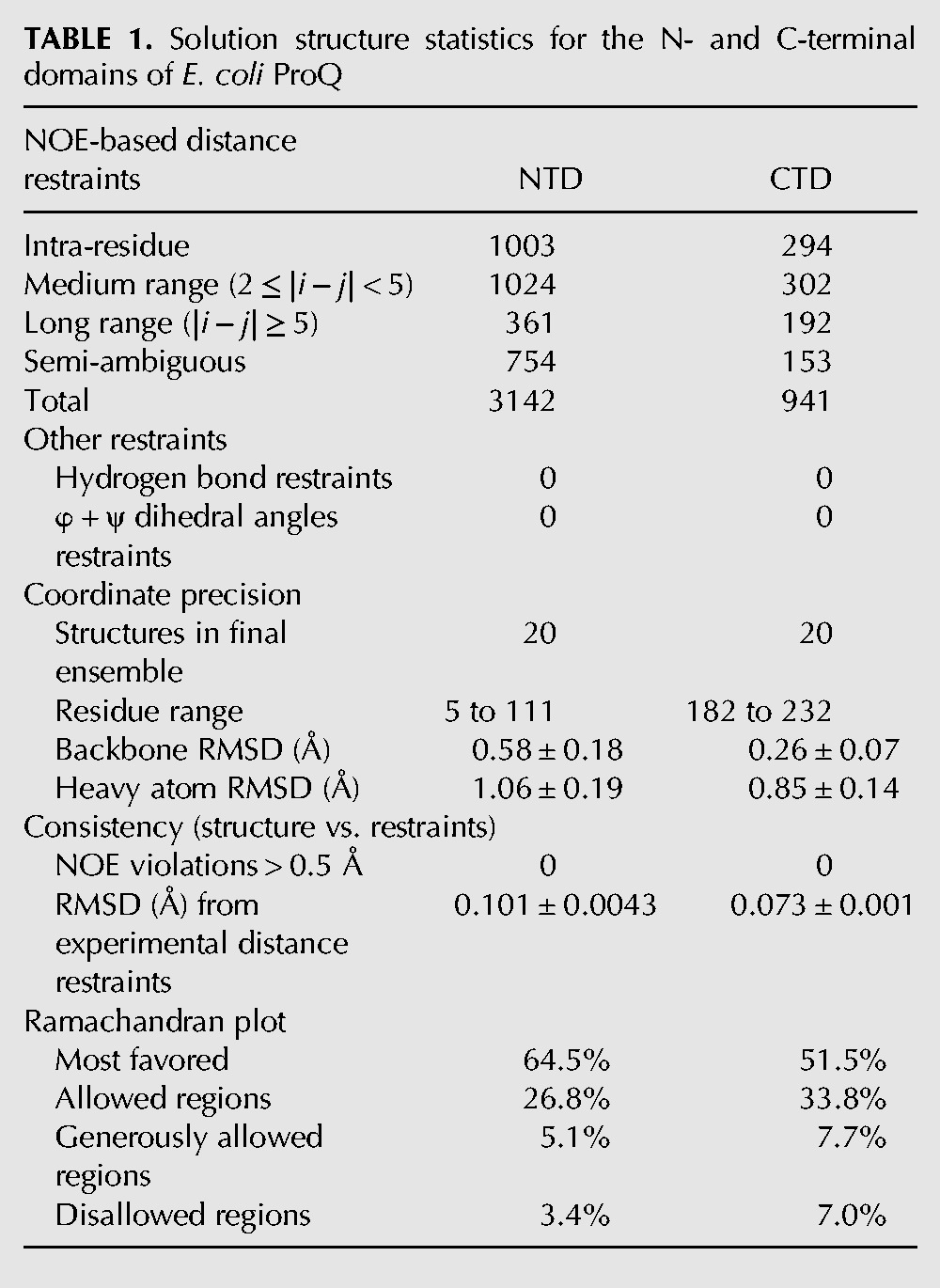
Solution structure statistics for the N- and C-terminal domains of *E. coli* ProQ

The surface of ProQ NTD is punctuated by interspersed patches of positive charge (Fig. 2B). Given that ProQ NTD functions as an RNA-binding protein (Chaulk et al. 2011), one might expect that a positively charged surface patch could be the site of RNA interaction. The electrostatic surface of the ProQ-NTD is described further in the discussion section of this article.

**FIGURE 3.**
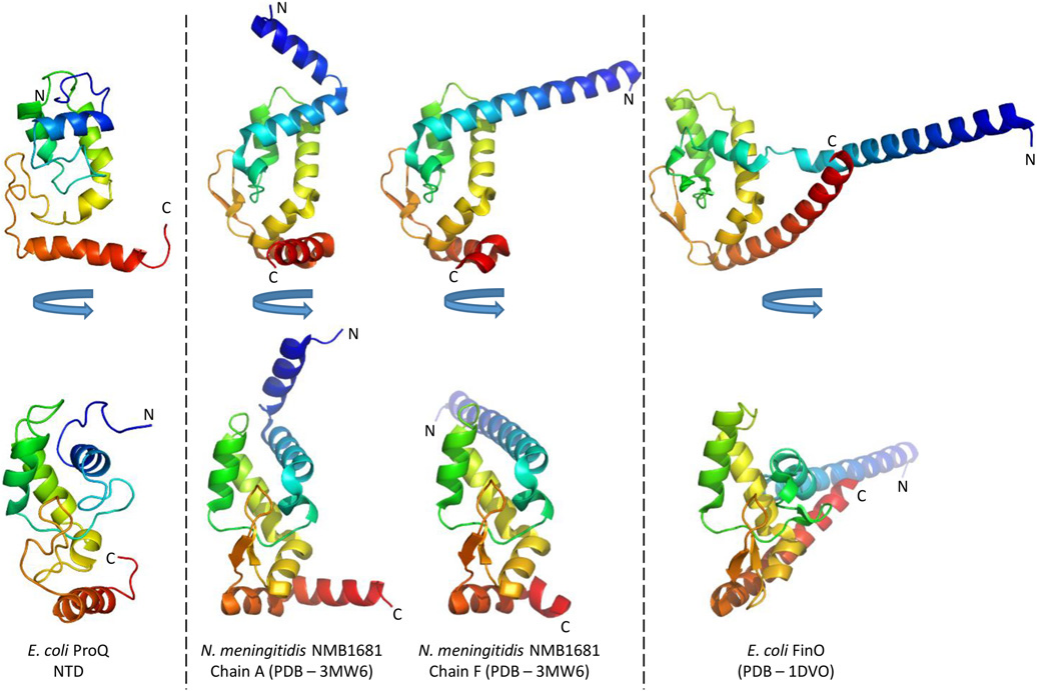
Structural comparison of the N-terminal domain of ProQ with other proteins. The lowest energy model of the *E. coli* ProQ NTD is shown in two orientations in cartoon representation colored as rainbow (blue–red, N-C termini). Chains A and F of *N. meningitidis* NMB1681, and *E. coli* FinO are shown in the same orientations, also colored as rainbow from blue to red as a comparison.

The ProQ NTD clearly shares structural homology with other ProQ/FinO domain proteins as reported in earlier studies (Ghetu et al. 2000; Chaulk et al. 2010), and the predominantly helical composition of this domain is in agreement with circular dichroism spectra (Fig. 2A; Smith et al. 2007). The similarity with ProQ/FinO domain structures was confirmed by the DALI protein structure comparison server (Holm and Rosenström 2010), which identified the closest structural match as being the ProQ/FinO domain protein NMB1681 of *N. meningitidis*, with a similarity *Z*-score of 5.6 and core RMSD of 2.51 Å (Chaulk et al. 2010), closely followed by *E. coli* FinO with a similarity *Z*-score of 4.4 and core RMSD of 2.68 Å (Ghetu et al. 2000). The structural similarity between the *E. coli* ProQ NTD, the equivalent domain from *N. meningitidis* NMB1681 and *E. coli* FinO is shown in Figure 3. The crystal structure of *N. meningitidis* ProQ contains six molecules in the crystallographic asymmetric unit, and the N-terminal α-helix is found to adopt alternative conformations in the different molecules present. In the NMR structure of the *E. coli* ProQ NTD, we observe that the 10 N-terminal amino acids corresponding to this α-helix adopt a single conformation, packed anti-parallel against α-helix one (residues 11–20) (Figs. 2A, 3).

### The NMR structure of the C-terminal domain of ProQ

**FIGURE 4.**
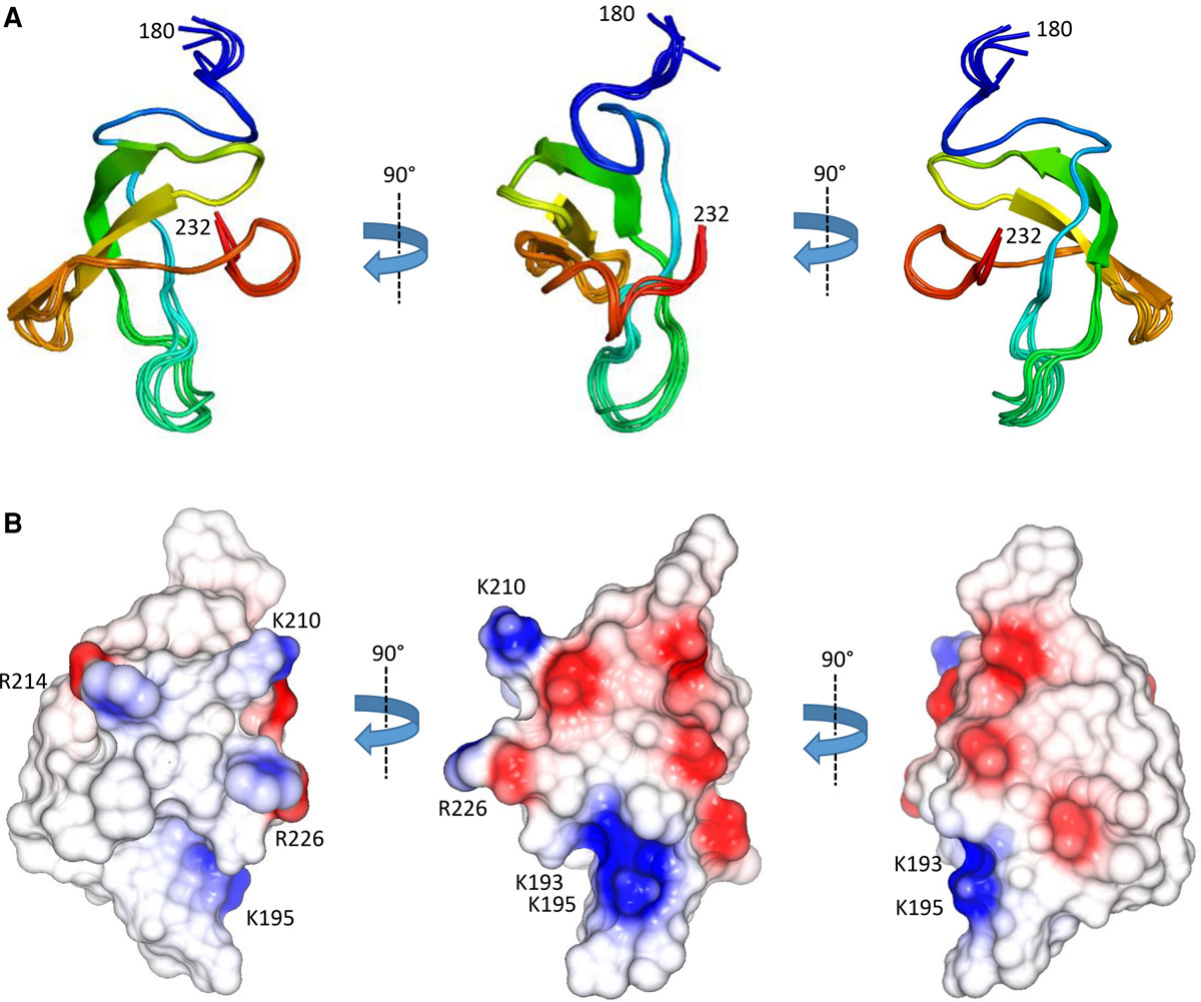
The NMR structure of the C-terminal domain of ProQ. (*A*) Ensemble of the top five solutions shown as cartoon representation and colored as rainbow from blue to red. (*B*) Electrostatic surface representation of the ProQ CTD (two orientations as in Fig. 4A). Charged surface exposed amino acids are labeled.

The structure of the C-terminal domain of ProQ (residues 180–232) was determined by NMR analysis of the full length ProQ protein. The structure calculations used a total of 941 NOE-derived distance restraints, of which 788 were unambiguously assigned. The final ensemble (Fig. 4A) has a backbone coordinate root mean square deviation (RMSD) of 0.26 Å over residues 182–232 (further statistics are summarized in Table 1). In agreement with previous CD spectroscopy studies on the ProQ CTD (Smith et al. 2007), the domain is comprised largely of β-strands (Fig. 4A). The surface of the ProQ CTD is predominantly electro-neutral, but there is some enrichment of electro-positive groups on one face and electro-negative groups on another face (left and right panels, respectively, in Fig. 4B).

**FIGURE 5.**
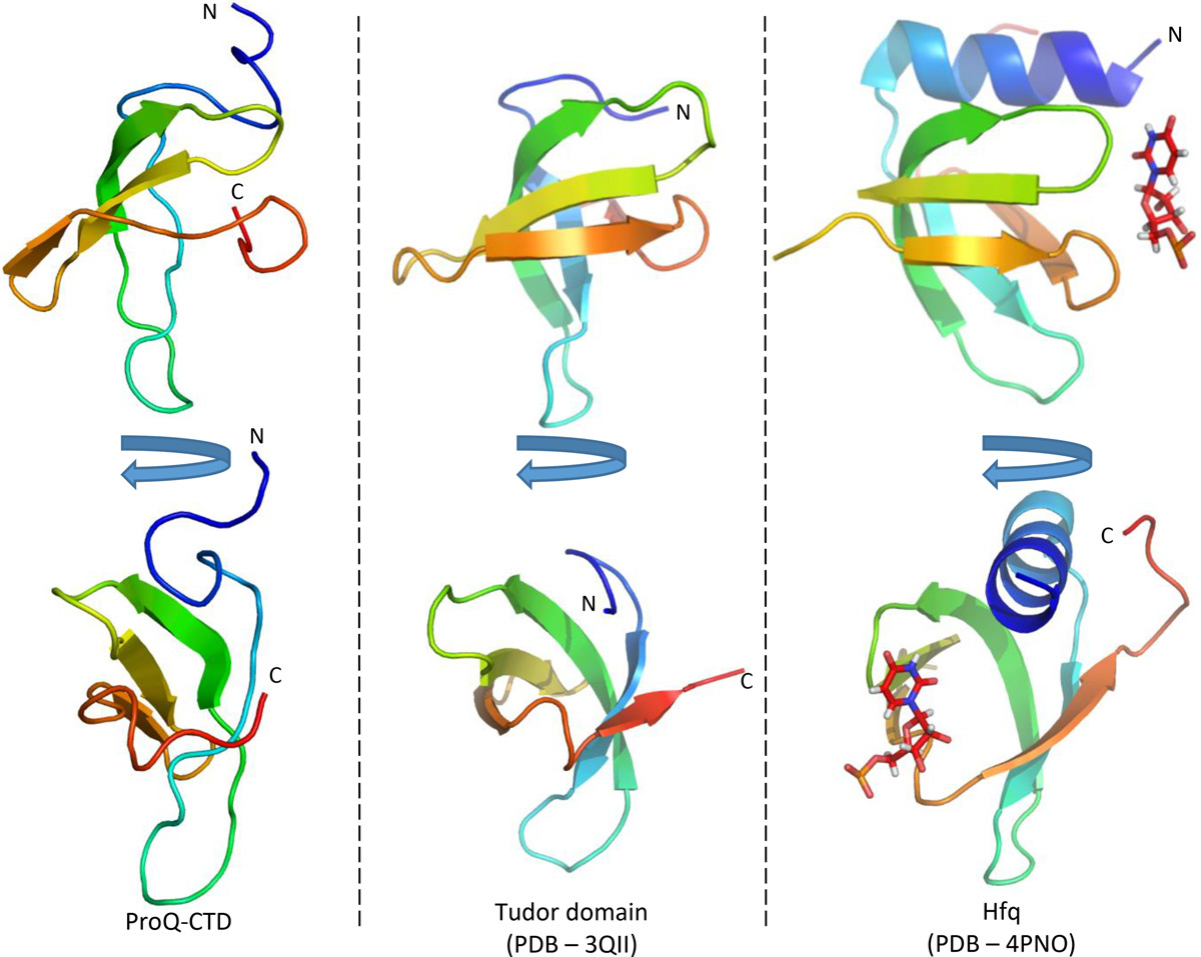
Structural comparison of the C-terminal domain of ProQ with other proteins. The lowest energy model of the ProQ CTD is shown in two orientations in cartoon representation colored in blue to red rainbow. Tudor domain 2 of human PHD finger protein 20 (PDB 3QII) and *E. coli* Hfq (PDB 4PNO) are shown in the same orientations, also colored as rainbow from blue to red. A UMP molecule bound in the Hfq model is shown as stick representation.

Next, we sought to clarify a previously predicted structural similarity between the CTD of ProQ and Hfq (Chaulk et al. 2011). While a visual comparison and a core RMSD of 2.04 Å following structural superimposition at first suggests that the domain may have a similar fold to the protomer of the hexameric *E. coli* Hfq (Fig. 5), a search for other structural homologs of the ProQ CTD using the DALI protein server (Holm and Rosenstrom 2010) returned multiple high-scoring hits for Tudor domain-containing proteins with superior matches. The top-scoring hit is Tudor domain 2 of human PHD finger protein 20 (PDB 3QII), with a similarity *Z*-score of 6.4 and core RMSD of 1.8 Å. The structure of Tudor domain 2 of human PHD finger protein 20 is compared with the ProQ CTD and *E. coli* Hfq in Figure 5. Tudor domains typically mediate protein–protein interactions through recognition of modified amino acids such as methylated lysine side chains. However, such modifications are not known to occur in bacteria. Indeed, the characteristic “aromatic cage” formed by aromatic residues at the methylated-ligand binding site is not conserved in the ProQ CTD, and is instead composed predominantly of hydrophobic residues, suggesting some alternative or divergent function.

Using the *E. coli* ProQ CTD sequence as a query for an iterative hidden Markov motif search (Jackhmmer webserver) (Finn et al. 2015), a match was identified to a protein from *Bacteroidetes bacterium* LOB10, which contains a central Tudor-knot domain in the Pfam database (UniProt: A0A136NFQ3_9BACT). Further searches of this C-terminal region using Jackhmmer identify this domain as belonging to the Agenet family group within the Tudor domain superfamily. These results support our proposal that the ProQ CTD is a distant member of the Tudor domain superfamily. A new domain family has been constructed for the C-terminal domain of ProQ and added to the Pfam database with accession number PF17516, and this family has also been added to the TUDOR domain superfamily (Pfam clan CL0049). Tudor domains are specific to eukaryotes, with Pfam reporting that of the 26,021 examples of Tudor domains only 39 (0.15%) are found in bacteria. This along with the finding that the CTD has a restricted phylogenetic distribution compared to all ProQ proteins may suggest that the CTD was acquired horizontally, most likely from a eukaryotic source.

### ProQ may exclude Hfq from binding to shared RNA targets

**FIGURE 6.**
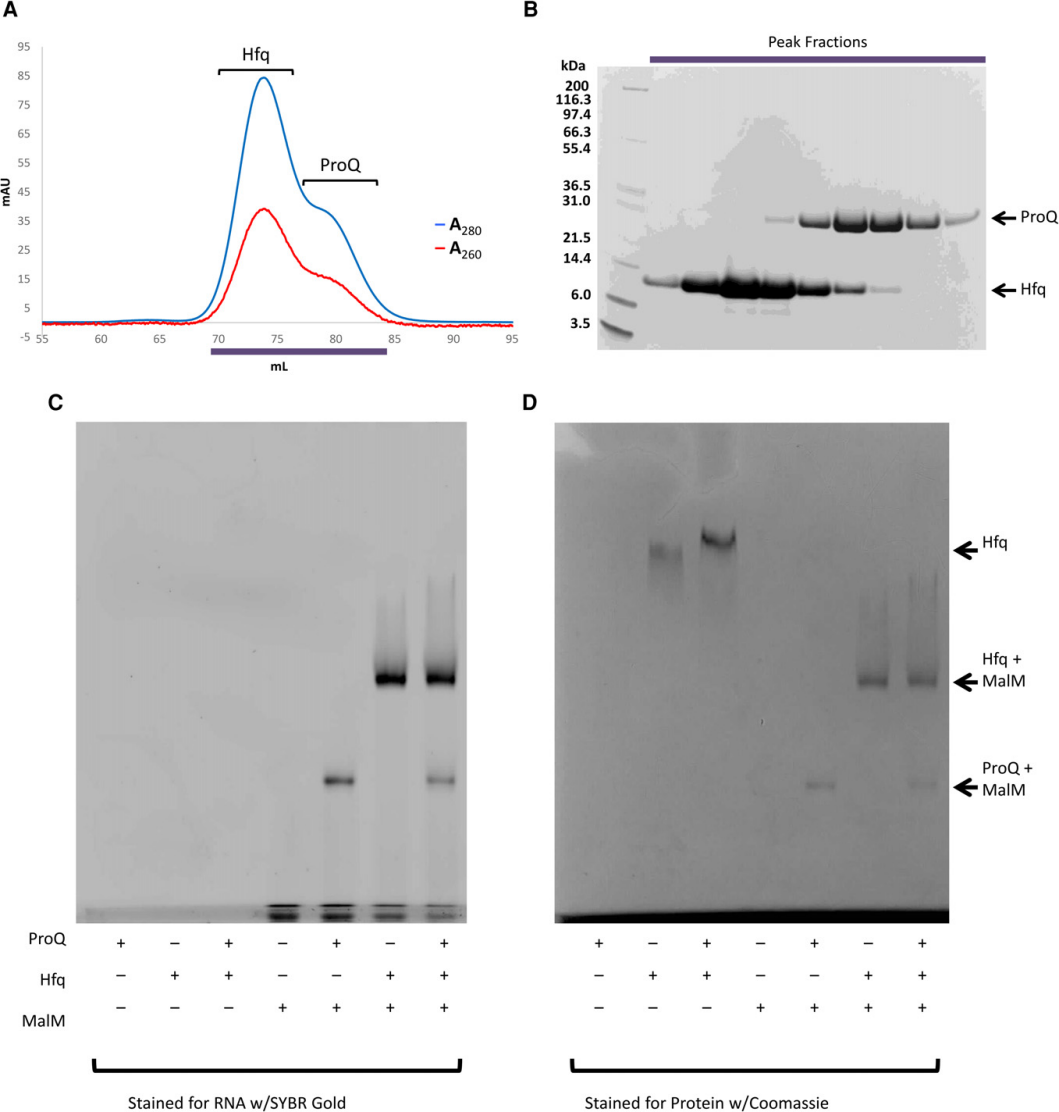
Hfq and ProQ do not physically interact directly and compete for interaction with the *malM* 3′ UTR. ProQ and Hfq were mixed at a 1:6 molar ratio and purified by gel filtration chromatography. The chromatogram is shown in *A*, and SDS–PAGE gel of the eluted fractions is shown in *B*. MalM 3′ UTR, ProQ, and Hfq were mixed in equimolar ratios, run on a native polyacrylamide gel, and stained for RNA (*C*) and protein (*D*). N.B. Full length apo-ProQ does not enter the native gel.

Some RNAs associated with ProQ are not exclusive targets of that protein and can also be bound by other RNA-binding proteins. One example is the *malM* 3′ UTR, which associates with both Hfq and ProQ via overlapping binding sites (Holmqvist et al. 2016; E Holmqvist, in prep.). Given their shared target, the question naturally arises whether ProQ might also interact with Hfq, either through a protein–protein interaction or an RNA-mediated interaction. To first test whether a protein–protein interaction occurs between the two sRNA binding proteins, equimolar amounts of ProQ and Hfq were mixed and run on a size exclusion chromatography column, but no complex was observed (Fig. 6A,B). In a native electrophoretic mobility shift assay (EMSA), ProQ alone does not enter the gel; however, a mixture of ProQ and Hfq does result in a modest shift of the band corresponding to Hfq (Fig. 6D). This may indicate a weak direct interaction between the two proteins, but given the absence of a stable complex following gel filtration chromatography we believe the altered migration of Hfq in the EMSA may be simply due to an altered pH of the sample in the presence of ProQ. To test whether RNA might bring the two proteins together, or if binding by one protein excludes the second from binding, ProQ, Hfq, and an equimolar mixture of the two was incubated with their mutual target *malM* 3′ UTR, and the resulting complexes were analyzed by EMSA. Both proteins can form binary complexes with *malM* 3′ UTR, but a ternary complex including ProQ, Hfq, and *malM* is not observed (Fig. 6C,D). This is perhaps not surprising as CLIPseq experiments show the individual binding sites for each protein overlap on the RNA (E Holmqvist, et al., in prep.), so presumably binding of one RNA-binding protein occludes binding of the other.

### Mapping of RNA-binding residues of ProQ

Results of EMSA indicate that *Salmonella* ProQ binds the sRNAs STnc2090 and SibA with dissociation constants in the nanomolar range (Smirnov et al. 2016). *E. coli* ProQ has been shown to bind with similar affinity to the 3′ UTRs of the transcript cspE, and CLIPseq shows that ProQ binds the 3′ UTRs of the transcript cspD (E Holmqvist et al., in prep.). The binding of ProQ to the 3′ UTRs of cspE or cspD was evaluated by SEC-MALS. Stable complexes were generated by mixing apo ProQ with a molar excess of RNA and purified by gel filtration prior to analysis by SEC-MALS. In both cases the results demonstrate that ProQ forms a 1:1 complex with the RNA partner (Supplemental Fig. S5).

**FIGURE 7.**
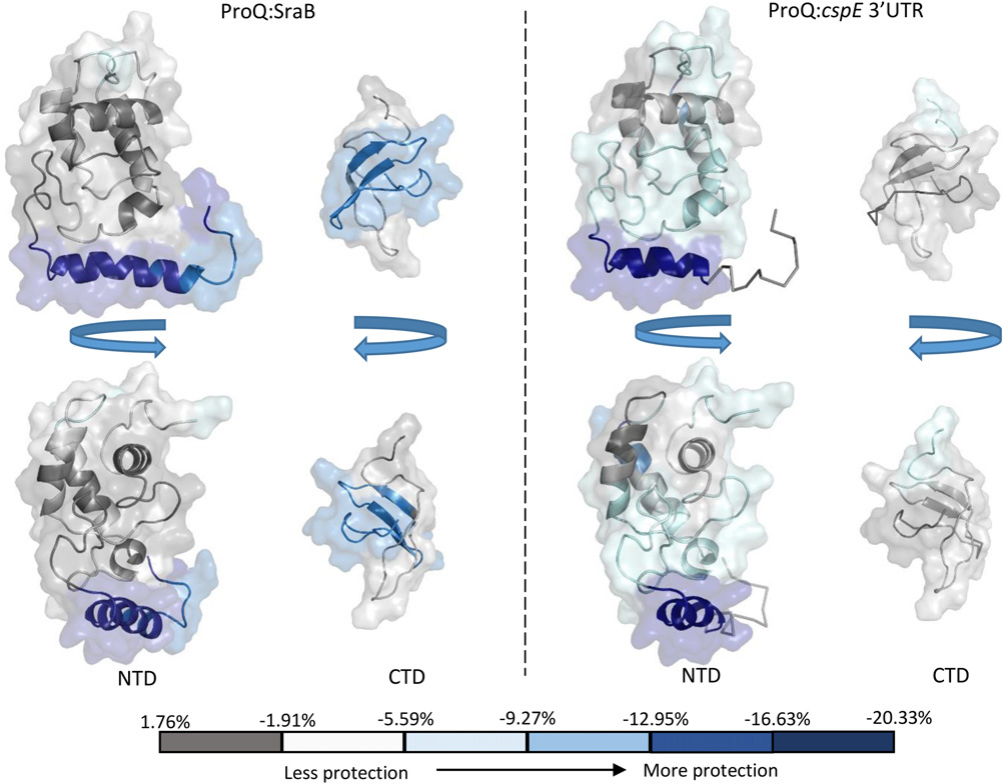
HDX mapping of RNA-binding surfaces on ProQ. Two views of the NMR models of ProQ N- and C-terminal domains are shown as cartoon representation with semitransparent surfaces. Regions protected upon binding to either SraB or *cspE* 3′ UTR in HDX experiments are colored with a heat-map (gray = less/no protection; blue = more protection). The boundaries between the heat-map divisions are shown on the bar at the *bottom*.

To further investigate the mechanism of sRNA binding by ProQ, mass spectrometry combined with hydrogen deuterium exchange experiments (HDX) was used to probe regions of ProQ that exhibit altered exchange profiles upon RNA binding. The sRNAs SraB and the *cspE* 3′ UTR were chosen, both of which are highly enriched from ProQ co-IP experiments (Smirnov et al. 2016). The two RNAs are predicted to be composed primarily of duplex regions, although the larger SraB (169 nt) consists of multiple shorter hairpins and *cspE* 3′ UTR (85 nt) of two longer hairpins. They do not have high sequence similarity and are representative of the likely structural diversity observed among the RNA targets of ProQ. The results from the HDX experiments are mapped as color-coded surfaces on the NMR structures of the N- and C-terminal domains of ProQ (Fig. 7) and peptide coverage maps for these experiments are shown in Supplemental Figures S6 and S7. These results imply that all three domains of ProQ can be involved in RNA binding, though it remains to be established what surfaces are directly interfacing with RNA and which may be merely remodeled as a consequence of binding elsewhere on the surface of the protein. Additionally, it is likely that different surfaces of ProQ may be used for different RNAs. Several patches of the FinO-like N-terminal domain, spanning residues 1–10 and 92–105, appear to be consistently protected from deuteration for both tested RNAs. The most highly protected conserved surface corresponds to residues 92–105, a region that overlaps with a basic patch on the protein surface formed of residues R32, R69, R80, R100, K101, K107, and R114, as seen in the electrostatic surface representation of ProQ in Figure 2 (left panel). In addition, the HDX data indicate residues 26–38 are involved in binding to *cspE* 3′ UTR but not significantly to SraB. Again, this region partially overlaps with the same electro-positive region highlighted in the left panel of Figure 2. Residues 184–203 of the Tudor-like C-terminal domain appear to be involved in RNA binding to some extent with both RNA substrates, but residues 205–216 of this domain show significant interaction with SraB but not *cspE* 3′UTR.

Despite limited HDX coverage for the linker region of ProQ, it does appear that this region is capable of binding SraB (Supplemental Fig. S6). As the HDX data suggest a greater role in binding to SraB than *cspE* 3′ UTR by the linker and C-terminal domains, it is possible that these regions only engage larger RNA substrates, whereas the NTD alone is sufficient for smaller substrates. The N-terminal domain appears to interact with RNAs via conserved surfaces (Fig. 7). These observations raise the interesting possibility for distinct roles for the NTD, linker, and the CTD. The NTD might be a specific RNA-binding anchor that brings the linker and the CTD in sufficient proximity to RNA targets for restructuring.

The results from the HDX experiments raised the possibility that the ProQ NTD alone may be sufficient to specifically bind RNA. In order to test this, an RNA-binding assay (Supplemental Fig. S8) was performed with known ProQ RNA targets and a nontarget to see if the NTD could discriminate between these different RNAs. The assay confirmed that the NTD can indeed discriminate among the presented RNAs. Shifts were observed for all confirmed RNA targets—*cspE* 3′ UTR, *cspD* 3′ UTR, and SraB sRNA—but not for the nontarget RNA, GlmZ, which is an Hfq-binding sRNA involved in amino-sugar regulation. GlmZ is also a highly structured RNA with a number of duplex regions (Göpel et al. 2014). It is unclear what makes it a nonbinder, but the NTD was, nonetheless, able to discriminate against GlmZ. This would suggest that the surfaces of the NTD involved in RNA binding are able to specifically identify motifs of ProQ-associated RNA targets.

### A molecular shape for full length ProQ

Previous analyses of ProQ by size exclusion chromatography have indicated an elongated conformation with a Stokes radius of 31 Å (Smith et al. 2004). In agreement with that finding, our analytical ultracentrifugation (AUC) data also indicate that ProQ has an elongated shape: The sedimentation coefficient of 1.72 S is consistent with a 25 kDa monomer with a high frictional coefficient of 1.67 (Supplemental Fig. S1D). Finally, our NMR relaxation data indicate that the N- and C-terminal domains do not interact to form a globular structure. Taken together this suggests that ProQ adopts an extended conformation in solution.

**FIGURE 8.**
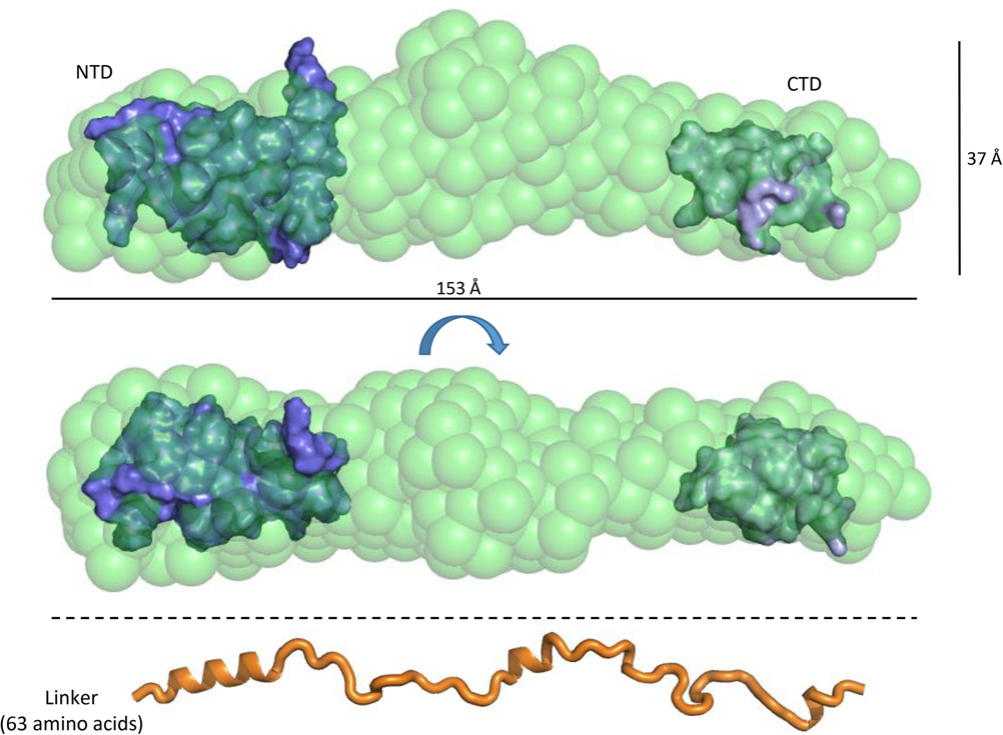
Solution shape of ProQ by small-angle X-ray scattering. (*Top*) The molecular envelope for ProQ is shown as semitransparent green spheres, with manually docked NMR models of the N- and C-terminal domains shown as solid blue surfaces. (*Bottom*) An extended 63 amino acid peptide is shown in scale to the *upper* panels to illustrate the potential distance the unmodeled linker region can span. The annotated sizes are direct measurements of the shown DAMMIF model. The average values from fitting the SAXS data for the *D*_max_ and *R*_g_ are 173.0 and 43.3 Å, respectively.

To assess the overall conformation, the full length ProQ protein was analyzed by SEC-SAXS (size exclusion chromatography combined with small-angle X-ray solution scattering). These data provide further evidence that ProQ is indeed highly extended, with a radius of gyration (*R*_g_) of 43.3 Å and a *D*_max_ value of 173.0 Å (Fig. 8; Supplemental Fig. S9). Ab initio modeling of *apo* ProQ produces a highly extended shape, with three bulges along the length of the rod that may correspond to the globular N- and C-terminal domains and the flexible linker region. Given all the biophysical evidence to indicate that ProQ adopts an elongated conformation in solution, with the N- and C-terminal domains behaving independently, we tentatively docked the NMR structures of the globular domains of ProQ into the SAXS envelope so that they occupy either ends of the rod. The remaining volume of the envelope would therefore be accounted for by the linker region of ProQ. As a visual reference, an extended polypeptide model of the ProQ linker region is shown at the bottom of Figure 8 to scale with the SAXS models above. Even allowing for a few regions of helical secondary structure, this model of the linker demonstrates that these 63 amino acids are long enough to bridge the gap between the N- and C-terminal domains. There is scope for the linker to compress to some extent to fit the space between the two globular terminal domains, but the semi-extended behavior of the linker is likely to arise from the striking pattern of charged residues (highlighted in Fig. 1B), which may cause internal repulsion that sustains the elongated structure.

### A molecular shape for a ProQ–RNA complex

**FIGURE 9.**
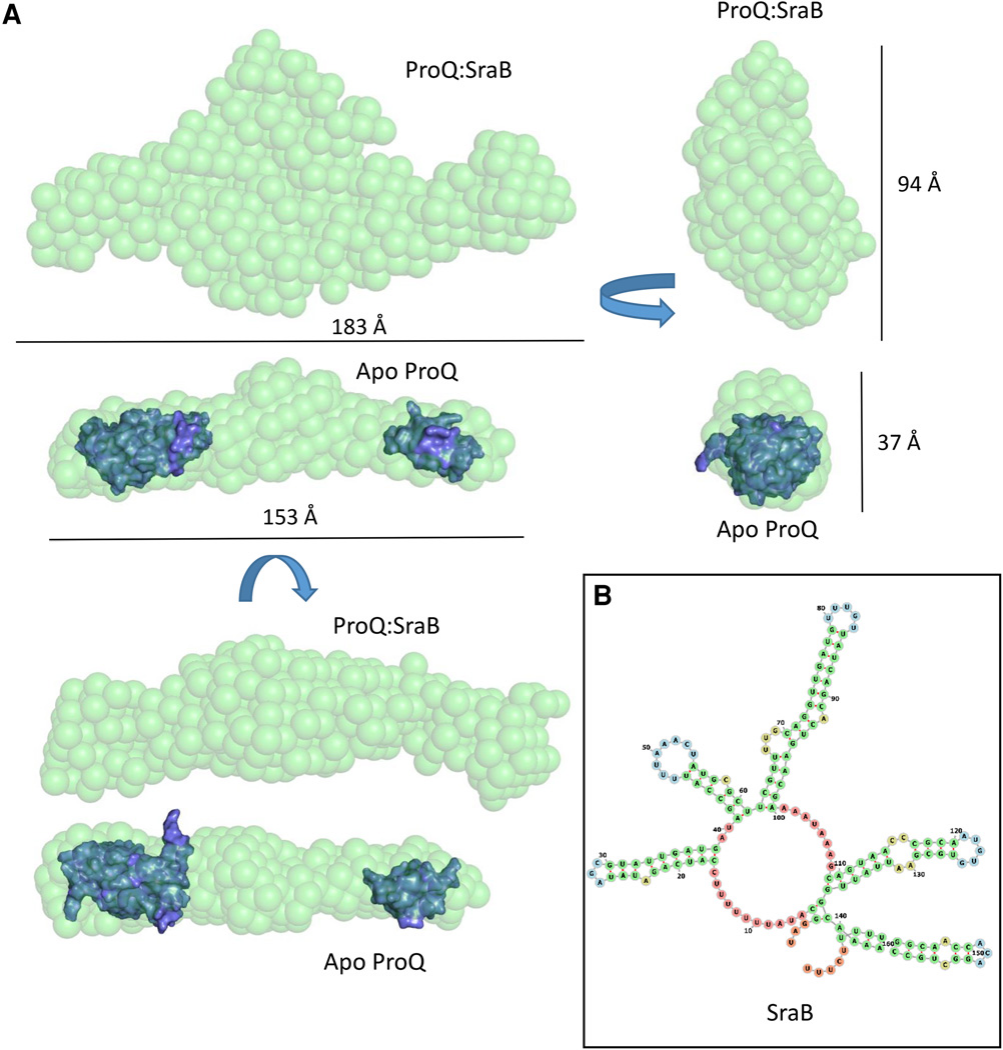
Solution shape of ProQ in complex with the sRNA SraB. (*A*) Three views of the molecular envelopes of ProQ:SraB and *apo* ProQ, shown as green spheres and to the same scale. The NMR models of the N- and C-terminal domains of ProQ are shown as blue surfaces and are manually docked into the SAXS envelope of *apo*-ProQ. (*B*) Secondary structural model of SraB, generated by RNAfold. The annotated sizes are direct measurements of the shown DAMMIF models. The average values for the *D*_max_ and *R*_g_ from fitting the ProQ:SraB SAXS data are 254.0 and 54.36 Å, respectively.

SEC-SAXS data were obtained for ProQ in complex with its cognate RNA partner SraB (Fig. 9; Supplemental Fig. 9). Data analysis revealed that, similarly to *apo* ProQ, the ProQ–SraB complex adopts an extended structure. In the presence of the RNA there is a significant increase in the calculated maximum diameter of the molecule from 173 to 254 Å, and upon inspection of the 3D envelope generated from the SAXS data the greatest increase in volume of the complex is around the central region presumed to be occupied by the linker in the apo ProQ model. The averaged ab initio models generated by DAMMIF (Franke and Svergun 2009) suggest that ProQ may not collapse in complex with RNA, but rather may maintain an extended structure that exposes RNA-binding surfaces to its target RNAs which, similarly, tend to be composed predominantly of long, duplex regions (Smirnov et al. 2016).

The SAXS model and HDX data both suggest that the central linker region may be implicated in binding the sRNA SraB. In support of this hypothesis, proteolytic digestion by trypsin of full length ProQ alone and in the presence of twice-molar excess of SraB reveal striking differences in the resulting products (Supplemental Fig. S10). In the absence of SraB, there appear to be only two major proteolytic products, consistent with the two globular domains being protected from internal cleavage and the linker being consumed. However, in the presence of SraB, the pattern of cleavage products changes and a significant amount of full length ProQ remains. These results suggest that binding of SraB masks potential trypsin cleavage sites within the linker region of ProQ, and may also indicate that the linker is not simply a flexible, interdomain tether, but also contributes to RNA binding.

**FIGURE 10.**
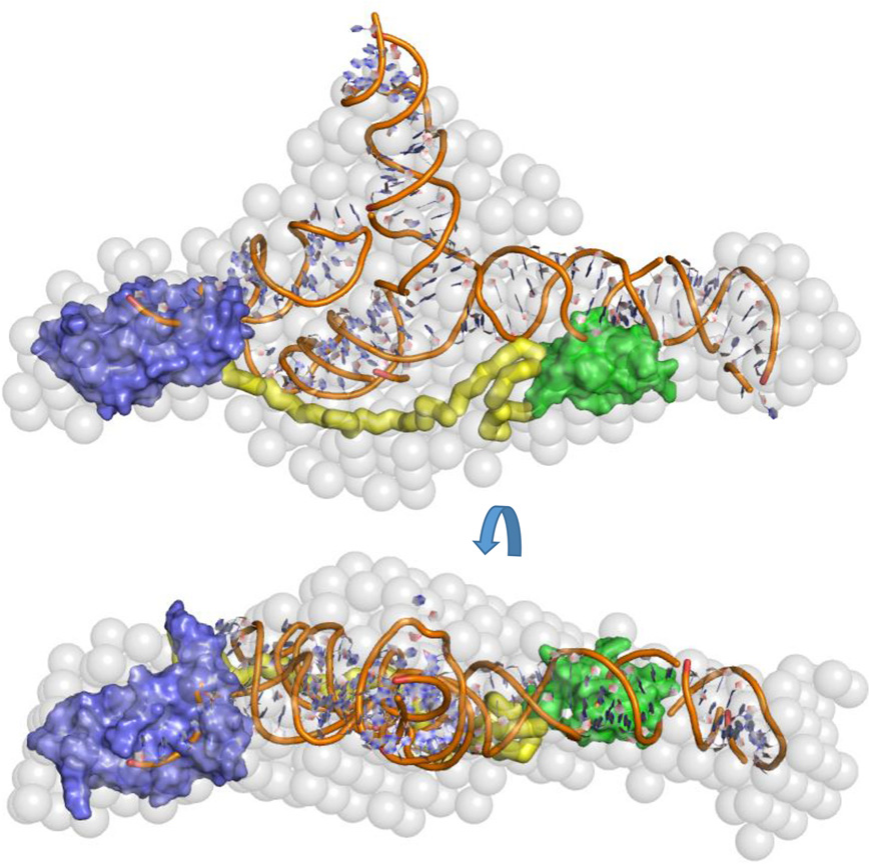
Docking of ProQ and SraB into the ProQ:SraB solution envelope. Two views of the solution envelope of ProQ:SraB (as in Fig. 7) shown as gray spheres, with manually docked NMR models of the ProQ N- and C-terminal domains shown as solid blue and green surfaces, respectively. A 63 amino acid extended peptide is shown as yellow ribbon to represent the ProQ linker region. A model of SraB generated by SimRNAweb with some helix positions rotated is manually docked into the SAXS envelope and is shown as cartoon representation.

The extended, elongated structure suggests that the N- and C-terminal domains are capable of interacting with spatially distant regions of the same RNA substrate. The RNA-binding potential of the N-terminal domain of ProQ and the disordered linker region have been previously described (Sheidy and Zielke 2013); however, RNA binding has not previously been attributed to the CTD. Taken together, our data indicate that all domains of ProQ contribute to RNA binding, at least for the SraB target. To provide a visual impression of how the ProQ domains and SraB might be arranged when in complex, a speculative model of SraB generated by SimRNAweb (Magnus et al. 2016) and the NMR models of the N- and C-terminal domains of ProQ are shown docked into the SAXS envelope of ProQ:SraB (Fig. 10).

## DISCUSSION

FinO-like RNA-binding proteins were previously considered to have only a few specific RNA targets. However, ProQ has recently been shown to act broadly as a global RNA-binding protein in *Salmonella*, and, in fact, has a similar number of RNA targets to the well-studied RNA chaperone protein Hfq (Smirnov et al. 2016). How such a small protein as ProQ can interact with so many different RNAs is an intriguing structural puzzle. The work presented here, taken together with several previous studies, provides clues into the basis of this molecular recognition. *E. coli* ProQ is monomeric, adopts an extended conformation, is composed of globular N- and C-terminal domains separated by a flexible linker, and all three domains may be used to interact with target RNA independently or cooperatively, depending on context. The NMR data corroborate that the *E. coli* ProQ NTD has the fold of the well-conserved ProQ/FinO domain. Our NMR model of the CTD of ProQ confirms that this domain shares a degree of similarity with Hfq, as predicted earlier, but has greater affiliation with the Tudor domain superfamily. Tudor domains are typically found in eukaryotic proteins, and are often involved in recognition of methylated amino acid ligands via an “aromatic cage” formed by side chains of tryptophan, tyrosine, and phenylalanine residues in a conserved motif. In the ProQ CTD, however, the aromatic cage residues are not conserved, indicating an alternative role for this domain.

Although the role of FinO-like domains in RNA binding has been well established, detailed molecular data on the mechanism of RNA recognition by these domains are not currently available. Through our structural and biophysical analyses we have been able to identify a positively charged surface patch on the NTD of ProQ that may be important for RNA binding. This region, delineated by residues R32, R69, R80, R100, K101, K107, and R114 (Fig. 2B, left panel) surrounds a surface patch identified on the homologous *L. pneumophila* RocC protein that is important for RNA binding and protein function (Attaiech et al. 2016). This positively charged surface patch also maps to a conserved protected region in our HDX experiments, indicating this area is important for RNA binding. On the opposite face of the molecule are more dispersed smaller electro-positive patches composed of K35, K54, R58, R62, K75, and R109 (Fig. 2B, center and right panels). RNA cross linking experiments have previously shown the residue corresponding to R58 as being implicated in RNA binding by FinO. Other residues implicated in RNA binding from this study of FinO clustered toward the N- and C-terminal helical extensions of this protein (Ghetu et al. 2002; Arthur et al. 2011).

The role of the CTD of ProQ is less well understood. Despite the widespread conservation of the FinO-like NTD in bacteria, the CTD appears to be present only as an appendage to ProQ of members of the gammaproteobacteria. Our HDX and SAXS data indicate that this domain does have a role in RNA binding, but potentially not with all substrates. The distributive interactions of the ProQ domains with RNA suggested by the HDX and EMSA data indicate that the domains can each interact with nucleic acid and may cooperate. This behavior may explain why deletion of individual domains may not totally abolish RNA binding in isolation (Sheidy and Zielke 2013).

Although there is no obvious conserved motif among ProQ-interacting RNAs, a common feature of sRNA substrates of ProQ is their propensity to form extensive duplex regions (Smirnov et al. 2016). Based on the SAXS and HDX data, a speculative model of the predominantly helical sRNA SraB bound to ProQ is shown in Figure 10. The extended form of ProQ found in the enterobacteria may have evolved as a mechanism to aid binding and protection of the extended duplex class of ProQ-binding sRNA molecules.

The question arises as to how ProQ interacts with so many structurally distinct RNAs. One possibility is that ProQ becomes recruited into multicomponent assemblies for which the interactions and specificities may be distributed among the components. The reported association of ProQ with the ribosome (Sheidy and Zielke 2013) and its presence in a super-complex including RNA polymerase, PNPase, and RNA (van Nues et al. 2016) could help to steer the preferred in vivo binding sites for ProQ. On the other hand, ProQ might also compete for binding sites with other proteins and chaperones, with important functional consequence. We observe that for *malM* 3′ UTR RNA, ProQ appears to compete with Hfq for a shared binding site on the RNA (Fig. 6). This suggests that there could be competition between sRNA binding proteins for RNA targets in vivo. This would then imply that the binding affinity and copy number of the respective proteins will be an important factor in dictating to which of these the potentially shared RNA molecule will bind.

This study provides a tentative view into the overall structure and functional relevance of ProQ's domain architecture, and it will be fruitful to fully probe the biological function of ProQ and assess bona fide sRNA chaperone activity. The previously reported association with such diverse systems as the ribosome (Sheidy and Zielke 2013) and a super-complex including RNA polymerase, PNPase, and RNA (van Nues et al. 2016) raise tantalizing possibilities for the biological function of ProQ. Might ProQ be involved in modulating the process of translation by affecting secondary structure features of its targets? Perhaps ProQ plays a role by inducing structural changes in 3′ UTRs to regulate termination. The structural data presented here may provide a framework for formulating and testing models for ProQ function.

## MATERIALS AND METHODS

### ProQ expression and purification

ProQ was amplified from genomic DNA isolated from *E. coli* strain K12 and ligated into a pET-DUET vector using primers ProQ_DUET2.F (CGCATATGGAAAATCAACCTAAGTTGAATAGC) and ProQ.R (GCCTCGAGTCAGAACACCAGGTGTTCTGCGCG). ProQ NTD (residues 1–119) was amplified from the plasmid expressing the full length protein using primers ProQ_DUET2.F and ProQ_119R (GCCTCGAGTCACGCTTGCTGTTCAGCATTGCA).

ProQ was expressed in BL21(DE3) cells grown in 2xYT media (Formedium) supplemented with 100 µg/mL carbenicillin at 37°C. Overnight precultures from a single colony in 2xYT media were diluted 2%–5% in fresh 2xYT-carbenicillin. Cultures were incubated at 37°C, 220 rpm, until the turbidity of the culture reached OD_600_ ∼ 0.4 and were induced with 1 mM IPTG. After 2 h, cells were then harvested by centrifugation at 4200*g* for 25 min at 4°C. The pellet was then resuspended in lysis buffer (20 mM Tris, pH 7.5, and Protease Inhibitor Cocktail Tablet [Roche]) and passed three times through an Emulsiflex-05 cell disruptor (10–15 kbar; Avestin).

The lysate was clarified with centrifugation at 37,500*g* for 30 min at 4°C and passed through a 0.45 µm filter (Millipore) before being loaded on an SP HP column (GE Healthcare) equilibrated with SP buffer A (20 mM Tris, pH 7.5). Protein was eluted by a gradient with 100% SP buffer B (20 mM Tris, pH 7.5, 1 M NaCl) and analyzed by SDS-PAGE. Fractions containing ProQ were pooled and diluted with SP Buffer A to achieve a final buffer composition of 20 mM Tris, pH 7.5, 100 mM NaCl. In order to remove contaminating nucleic acid, the sample was then loaded onto a Heparin HiTrap HP column (GE Healthcare) equilibrated in Heparin Buffer A (20 mM Tris, pH 7.5, 100 mM NaCl). Protein was eluted by a gradient of Heparin Buffer B (20 mM Tris, pH 7.5, 1 M NaCl) and analyzed by SDS-PAGE.

Fractions containing pure *apo* ProQ were pooled, concentrated to 2 mL with 15 mL Amicon Ultra 10,000 MWCO concentrator (Millipore) and loaded onto a Sephadex 75 gel filtration column (GE Healthcare) equilibrated with buffer containing 50 mM Tris pH 7.5, 100 mM NaCl, and 100 mM KCl. Fractions were analyzed by SDS-PAGE and the concentration of ProQ in the fractions was determined spectroscopically using a NanoDrop ND-1000 spectrophotometer (Thermo Scientific) and a λ_280nm_ extinction coefficient of 9970 M^−1^cm^−1^ before they were flash frozen and stored at −80°C.

### Expression and purification of ProQ for NMR

Uniformly ^15^N or ^13^C/^15^N labeled proteins were produced using nitrogen-deficient phosphate buffered media, wherein nitrogen was supplied by ^15^NH_4_Cl. Where necessary, glucose was replaced with ^13^C-incorporated glucose. Protein was expressed in BL21(DE3) cells at 25°C for 20 h after induction with 1 mM IPTG. Cells were harvested and protein purified as described above. Purified ProQ was then concentrated to 0.75 mM.

### Expression and purification of ProQ NTD (residues 1–119) for NMR

Uniformly ^15^N or ^13^C/^15^N labeled proteins were produced using nitrogen- and/or glucose-deficient phosphate buffered media, as described above. Protein was expressed in BL21(DE3) cells at 25°C for 20 h after induction with 1 mM IPTG. Cells were harvested and protein purified as described above. Purified ProQ NTD was then concentrated to 0.70 mM.

### Large-scale RNA synthesis

RNA was produced by in vitro transcription (IVT). DNA template concentrations were varied from 1–2 µg/mL and T7 polymerase varied from 0.1–0.2 mg/mL. Individual rNTP concentrations were kept constant at 5 mM. Full-scale transcription reactions (200 µL) were carried out in T7 buffer (40 mM Tris, pH 8.0, 25 mM MgCl_2_, 0.01% Triton X-100, 0.2 mM Spermidine, and 10 mM DTT) and incubated at 37°C for 4 h, then DNA template was digested by Turbo DNaseI (Invitrogen), and the reactions were quenched with EDTA. RNA DNA templates for IVT were generated via ligation of two complementary DNA oligos encoding first the T7 polymerase promoter sequence (5′-GAAATTAATACGACTCACTATA) and then the sequence of the desired RNA. Ligation was achieved by mixing equimolar amounts of each oligo in Oligo Binding Buffer (10 mM Tris, pH 8.0, 50 mM NaCl, 1 mM EDTA), which was then heated at 95°C for 5 min on a heat block and left on the heat block to cool slowly to room temperature. Products of IVT reactions were purified on 8% polyacrylamide gels with 7.5 M urea. Bands containing RNA were visualized by UV-shadowing (254 nm) and excised. RNA was then recovered from the gel slices by overnight electroelution at 100 V in TBE buffer using an EluTrap System (Whatman).

### Size exclusion chromatography—small-angle X-ray scattering (SEC-SAXS)

SAXS experiments were performed on the SWING beamline at SOLEIL with a wavelength of 1.003 Å. The 17 × 17 cm^2^ low-noise Aviex CCD detector was positioned at a distance of 1800 mm, covering a *q* range of 0.004 < *Q* < 0.61 Å (where *q* is the scattering vector [4πsinθ/λ]). All samples were first prepared on a Superdex 200 Increase 3.2/300 column (GE Healthcare) in the same buffer used on the beamline. Samples were concentrated using Amicon Ultra 0.5 mL 5 kDa centrifugal concentrators. Fifty microliters of sample in SAXS buffer (50 mM Tris, pH 7.5, 200 mM NaCl, 5% [v/v] glycerol, and 1 mM TCEP) was injected into a Superdex 200 Increase 3.2/300 column using a beam line-integrated Agilent HPLC maintained at 20°C and eluted directly into the SAXS flow through capillary cell at a flow rate of 0.075 mL/min. SAXS data were collected online throughout the whole elution time, with a frame duration of 2 sec and a dead time between frames of 1 sec. A first data set of 90 frames, collected before the void volume, was averaged to account for buffer scattering. A second data set was collected for the sample, from which frames corresponding to the top of the elution peak were averaged and used for data processing after baseline subtraction.

Data were processed using the SWING beam line dedicated application, FOXTROT. Scattering curves were buffer subtracted and merged using Primus software (Konarev et al. 2003). At low angles, the *R*_g_ was found using the Guinier approximation, *I*(*q*) = exp(*R*_g_^2^*q*^2^/3), with *I*(0) indicating forward scattering intensity. Transformation of the scattering curve by the GNOM program (Svergun 1999) generated a distribution of particle distances, allowing the maximum dimension to be determined, *D*_max_. Confirmation of correct dimensions was achieved when the *R*_g_ from GNOM matched that obtained from the Guinier approximation. DAMMIF was used to make low-resolution ab initio models of the SAXS data (Franke and Svergun 2009). Twenty models were generated, averaged by DAMAVER, and filtered with DAMFILT to make a model that represented the most probable conformation (Volkov and Svergun 2003; Bernado and Svergun 2014; Tria et al. 2015).

### NMR experiments for assignment and distance restraints

All samples for nuclear magnetic resonance (NMR) spectroscopy were prepared at concentrations of 400–800 µM in buffer containing 20 mM sodium phosphate and 100 mM sodium chloride, supplemented with 1 mM TCEP, protease inhibitor (Roche, 1 tablet/l), 10% D_2_O (Sigma) and 0.0025% 3,3,3-trimethylsilylpropionate (Sigma-Aldrich), in 5 mm Ultra-Imperial grade NMR tubes (Wilmad) to a final volume of 550 µL. For backbone assignment, [^1^H,^15^N]-HSQC, ^15^N-separated TOCSY-HSQC, HNCA, HNCOCA, HNCACB, and CBCA(CO)NH spectra were recorded; for side-chain assignment and collection of distance restraints, [^1^H,^13^C]-HSQC, HCCH-TOCSY, ^15^N-separated NOESY-HSQC and ^13^C-separated NOESY-HSQC spectra were acquired. All experiments were collected at 298 K on Bruker DRX500, AV600, and AV800 spectrometers, using standard procedures (Cavanagh et al. 2006); the AV600 and AV800 spectrometers were each equipped with a 5 mm TXI CryoProbe. All NMR spectra were processed using the Azara package (www.ccpn.ac.uk/azara), then analyzed and assigned using CcpNmr Analysis software (Vranken et al. 2005).

### Determination of solution structures for the amino- and carboxy-terminal domains of ProQ

All structures of ProQ NTD and CTD were calculated from extended templates by simulated annealing using ARIA 2.3 (Bardiaux et al. 2009), with manual screening of ambiguous restraints. Backbone φ and ψ dihedral angle restraints were determined from chemical shifts using the DANGLE program (Cheung et al. 2010). NOE distance restraints generated by the resonance assignment process and dihedral angle restraints were fed as input. Nine iterations were performed, each using 20 structures, except for the final round, in which 50 were calculated, followed by refinement in explicit solvent for the 20 lowest energy structures, all of which were selected for the final ensemble, which contains no distance violations >0.5 Å and includes >97% of residues in the “most favored” and “allowed” regions of the Ramachandran plot. The atomic coordinates of the final ensemble for ProQ NTD and CTD were deposited in the Protein Data Bank under ID codes 5NB9 and 5NBB, respectively; the corresponding NMR assignments were deposited in the Biological Magnetic Resonance Data Bank under accession code 34110.

### ^15^N nuclear spin relaxation experiments

^15^N nuclear spin relaxation experiments were recorded using standard procedures (Cavanagh et al. 2006) at 298 K on a Bruker DRX500 spectrometer. ^15^N T_1_ delays (msec) were 10, 50, 100, 150, 250, 400, 550, 700, 850, 1000. ^15^N T_2_ delays (msec) were 14.4, 28.8, 43.2, 57.6, 72.0, 86.4, 100.8, 155.2. Heteronuclear NOE reference and saturation experiments were carried out in duplicate to allow an estimation of the error. The relaxation parameters were analyzed with version 3.1 of the ROTDIF program (Berlin et al. 2014).

### Hydrogen–deuterium exchange mass spectrometry (HDX-MS)

Deuterium exchange reactions of ProQ were initiated by diluting the protein in D_2_O (99.8% D_2_O ACROS, Sigma-Aldrich) in 25 mM Tris, 100 mM NaCl, 100 mM KCl, 10 mM MgCl_2_, 1 mM TCEP, pH 7.5 buffer to give a final D_2_O percentage of 90%. For all experiments, deuterium labeling was carried out at 23°C (unless otherwise stated) at four time points (3 sec on ice [0.3 sec], 3 sec, 30 sec, and 300 sec in triplicate). The labeling reaction was quenched by the addition of chilled 2.4% v/v formic acid in 2 M guanidinium hydrochloride and immediately frozen in liquid nitrogen. Samples were stored at −80°C prior to analysis.

The quenched protein samples were rapidly thawed and subjected to proteolytic cleavage by pepsin followed by reversed phase HPLC separation. Briefly, the protein was passed through an Enzymate BEH immobilized pepsin column, 2.1 × 30 mm, 5 µm (Waters) at 200 µL/min for 2 min and the peptic peptides trapped and desalted on a 2.1 × 5 mm C18 trap column (Acquity BEH C18 Van-guard pre-column, 1.7 µm, Waters). Trapped peptides were subsequently eluted over 12 min using a 5%–36% gradient of acetonitrile in 0.1% v/v formic acid at 40 µL/min. Peptides were separated on a reverse phase column (Acquity UPLC BEH C18 column 1.7 µm, 100 mm × 1 mm [Waters]). Peptides were detected on a SYNAPT G2-Si HDMS mass spectrometer (Waters) acquiring over a *m*/*z* of 300 to 2000, with the standard electrospray ionization (ESI) source and lock mass calibration using [Glu1]-fibrino peptide B (50 fmol/µL). The mass spectrometer was operated at a source temperature of 80°C and a spray voltage of 2.6 kV. Spectra were collected in positive ion mode.

Peptide identification was performed by MS^e^ (Silva et al. 2005) using an identical gradient of increasing acetonitrile in 0.1% v/v formic acid over 12 min. The resulting MS^e^ data were analyzed using Protein Lynx Global Server software (Waters) with an MS tolerance of 5 ppm. Mass analysis of the peptide centroids was performed using DynamX sotware (Waters). Only peptides with a score >6.4 were considered. The first round of analysis and identification was performed automatically by the DynamX software; however, all peptides (deuterated and nondeuterated) were manually verified at every time point for the correct charge state, presence of overlapping peptides, and correct retention time. Deuterium incorporation was not corrected for back-exchange and represents relative, rather than absolute changes in deuterium levels. Changes in H/D amide exchange in any peptide may be due to a single amide or a number of amides within that peptide. All time points in this study were prepared at the same time and individual time points were acquired on the mass spectrometer on the same day.

### Sequence analysis

Jackhmmer searches were performed against the UniProtKB database using the HMMER webserver with default parameters.

## SUPPLEMENTAL MATERIAL

Supplemental material is available for this article.

## Supplementary Material

Supplemental Material
