# Supplementary material for: Structure of the Escherichia coli ProQ RNA-binding protein
Source: RNA. 2017 May;23(5):696–711. doi: 10.1261/rna.060343.116 (PMC5393179; doi:10.1261/rna.060343.116)
Supplement: Supplemental Material [file supp_23_5_696__index.html]

Structure of the Escherichia coli ProQ RNA-binding protein — Supplemental Material 

# Structure of the *Escherichia coli* ProQ RNA-binding protein

## Supplemental Material

- Supplemental\_Fig\_S1.pdf
- Supplemental\_Fig\_S2.pdf
- Supplemental\_Fig\_S3.pdf
- Supplemental\_Fig\_S4.pdf
- Supplemental\_Fig\_S5.pdf
- Supplemental\_Fig\_S6.pdf
- Supplemental\_Fig\_S7.pdf
- Supplemental\_Fig\_S8.pdf
- Supplemental\_Fig\_S9.pdf
- Supplemental\_Fig\_S10.pdf
- Supplemental\_Figure\_Legends.docx
