# Supplementary material for: Structure of the Escherichia coli ProQ RNA-binding protein
Source: RNA. 2017 May;23(5):696–711. doi: 10.1261/rna.060343.116 (PMC5393179; doi:10.1261/rna.060343.116)
Supplement: Supplemental Material [file supp_060343.116_Supplemental_Fig_S1.pdf]

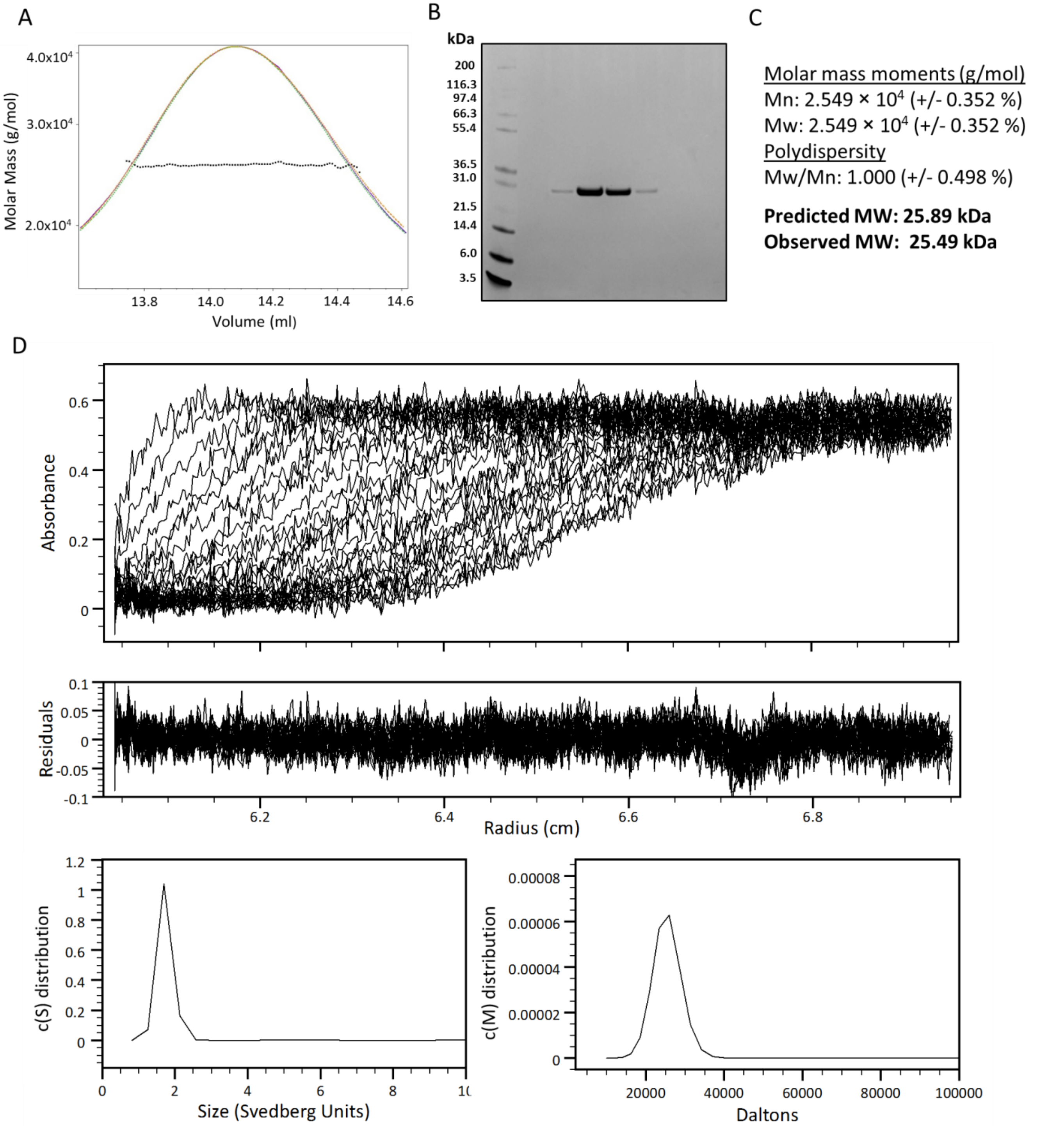


**Figure S1.** SEC-MALS and Analytical ultracentrifugation analysis of ProQ. A) SEC MALS elution profile showing estimated molecular mass variation over the elution profile. B) SDS-PAGE analysis of the peak from the SEC-MALS profile, demonstrating the purity of the sample. C) Summary of molecular mass estimates (Mn – number averaged, Mw- weight averaged) and polydispersity. D) AUC analyses - the velocity sedimentation profile and residuals are shown in the upper panels, and distribution plots as Svedberg units and Daltons are shown in the lower panels.
