# Supplementary material for: Structure of the Escherichia coli ProQ RNA-binding protein
Source: RNA. 2017 May;23(5):696–711. doi: 10.1261/rna.060343.116 (PMC5393179; doi:10.1261/rna.060343.116)
Supplement: Supplemental Material [file supp_060343.116_Supplemental_Fig_S3.pdf]

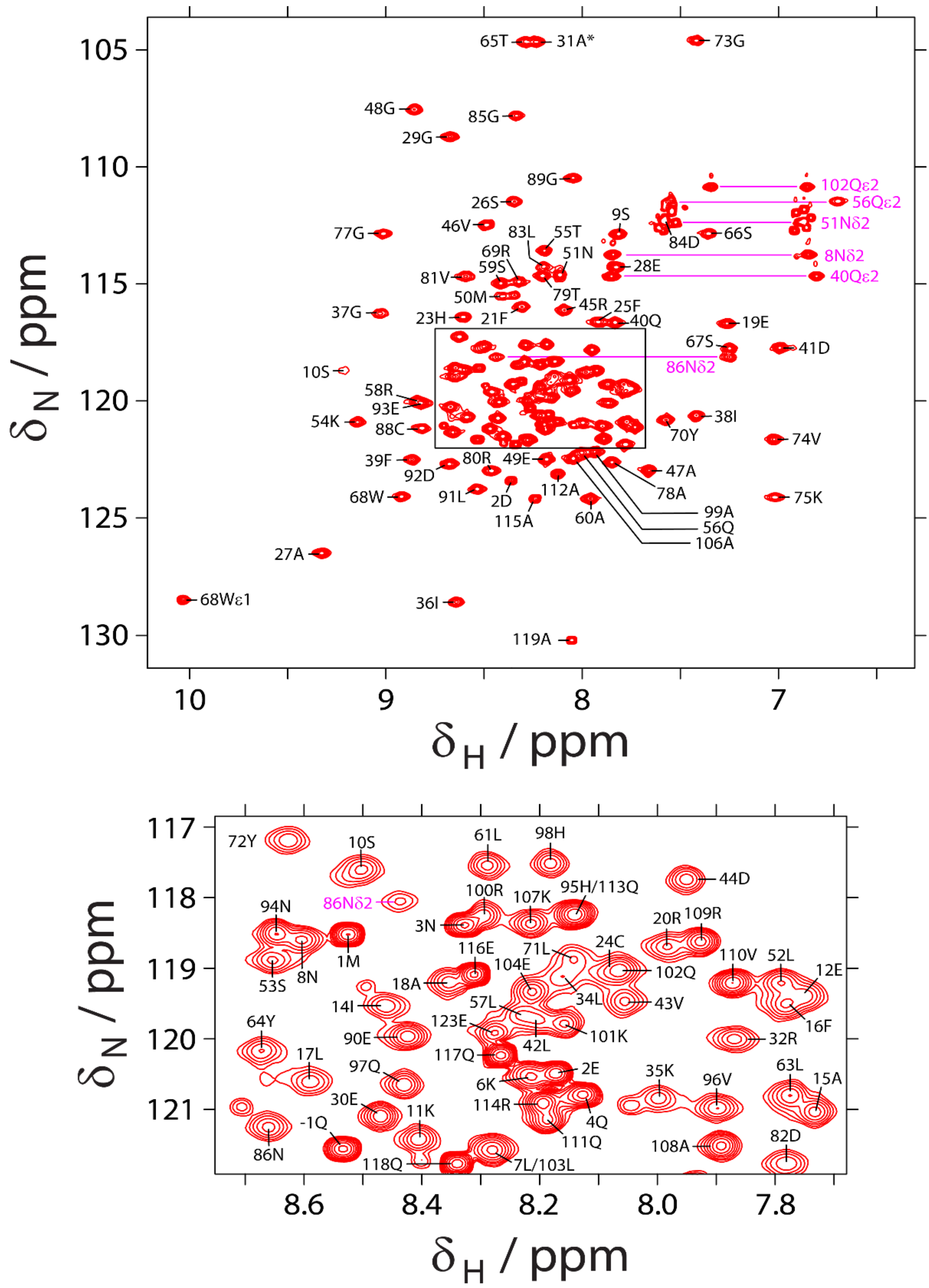

Figure S3. NMR data for ProQ-NTD. (Top) [^{1}H,^{15}N]-HSQC spectrum of ProQ-NTD, showing residue assignments for backbone amide sites. Pairs of resonances from side-chain amide sites are connected using magenta lines. (Bottom) Assignments for the closely spaced signals contained in the box shown in the upper panel.
