# Supplementary material for: Structure of the Escherichia coli ProQ RNA-binding protein
Source: RNA. 2017 May;23(5):696–711. doi: 10.1261/rna.060343.116 (PMC5393179; doi:10.1261/rna.060343.116)
Supplement: Supplemental Material [file supp_060343.116_Supplemental_Fig_S4.pdf]

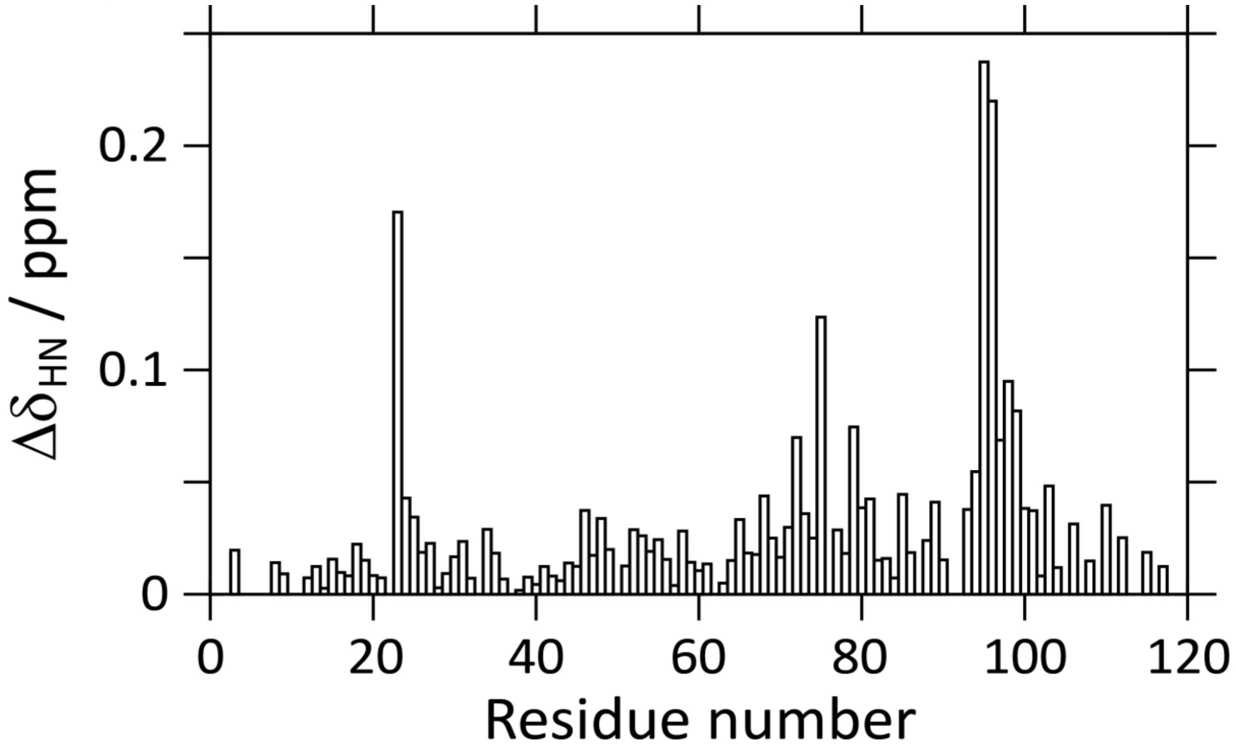

Figure S4. Histogram showing average HN chemical shift differences between the full length and NTD samples of ProQ plotted as a function of residue number. Average shift differences were determined using the equation $\Delta\delta_{HN} = \sqrt{(\Delta\delta_H^2 + 0.15\times\Delta\delta_N^2)}$.
