# Supplementary material for: Structure of the Escherichia coli ProQ RNA-binding protein
Source: RNA. 2017 May;23(5):696–711. doi: 10.1261/rna.060343.116 (PMC5393179; doi:10.1261/rna.060343.116)
Supplement: Supplemental Material [file supp_060343.116_Supplemental_Fig_S5.pdf]

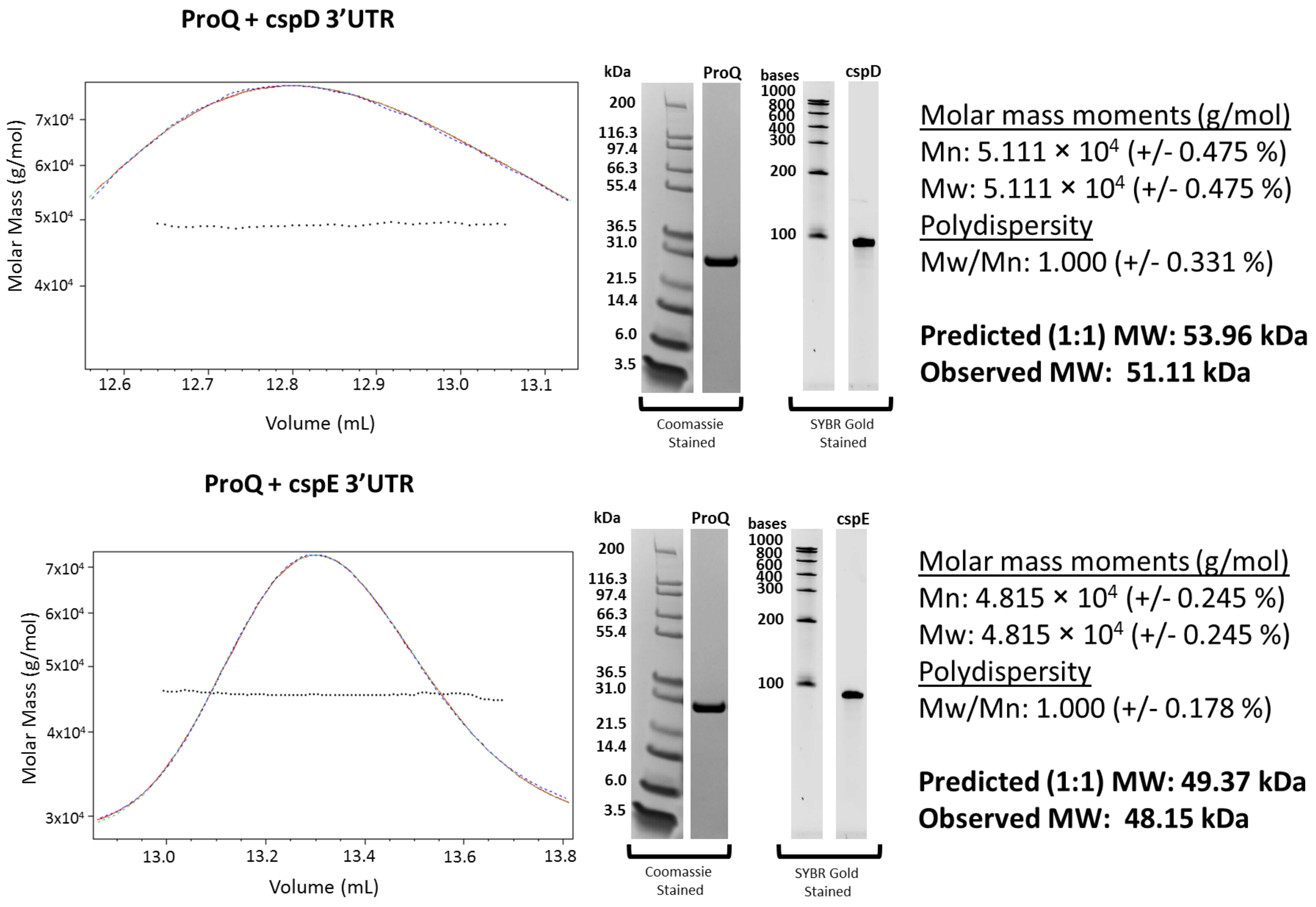

Figure S5. ProQ binds full length and truncated target RNA ligands at a 1:1 ratio. SEC-MALS analysis of ProQ with sRNAs derived from the 3'UTR of *cspD* (top) and *cspE* (bottom). Left panels - SEC MALS elution profile showing estimated molecular mass variation over the elution profile. Middle panels - SDS-PAGE analysis of the peak from the SEC-MALS profile, demonstrating the purity of the sample. Right panels - Summary of molecular mass estimates (Mn – number averaged, Mw- weight averaged) and polydispersity.
