# Supplementary material for: Structure of the Escherichia coli ProQ RNA-binding protein
Source: RNA. 2017 May;23(5):696–711. doi: 10.1261/rna.060343.116 (PMC5393179; doi:10.1261/rna.060343.116)
Supplement: Supplemental Material [file supp_060343.116_Supplemental_Fig_S7.pdf]

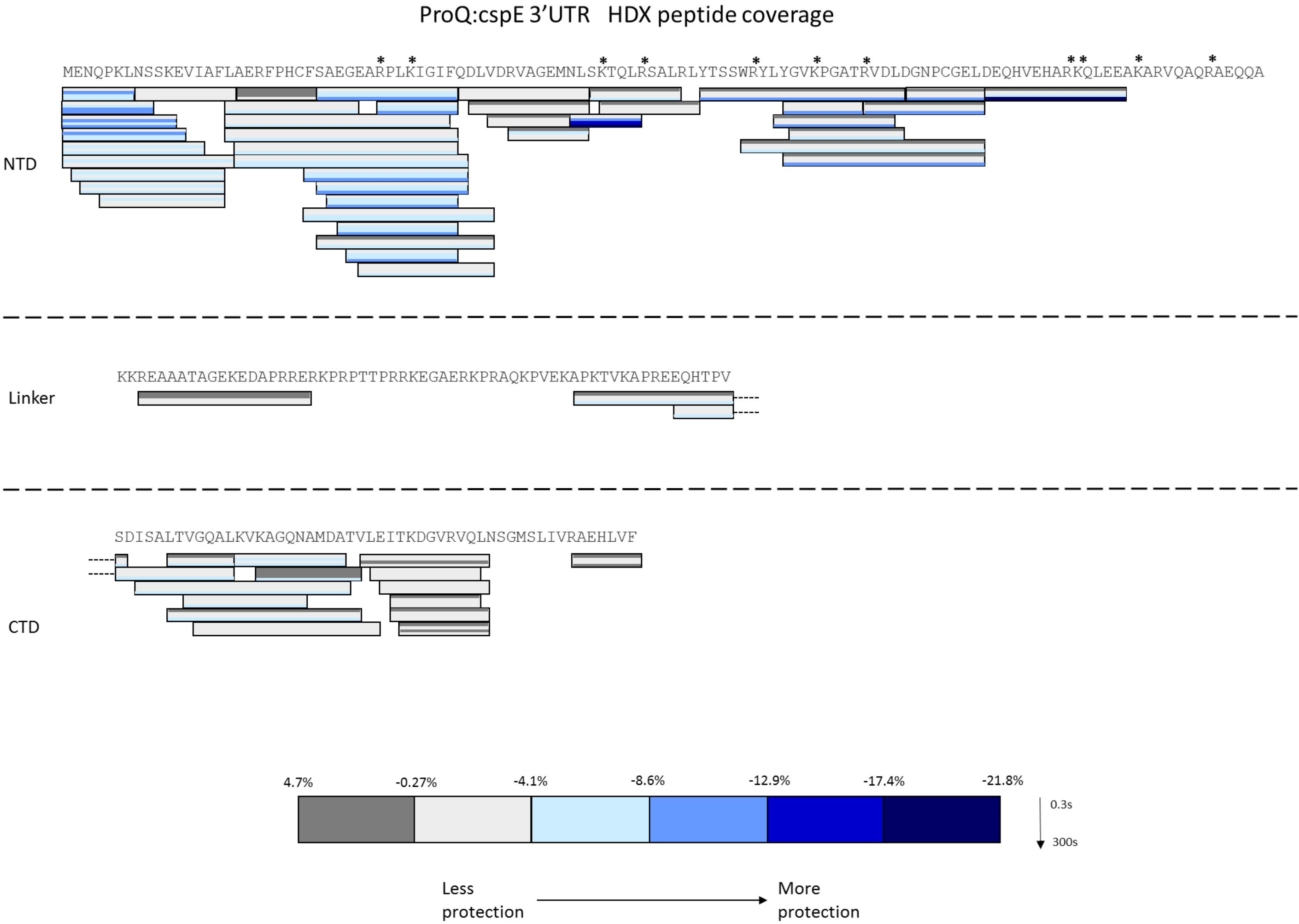

Figure S7. Peptide coverage for ProQ:cspE 3'UTR HDX experiment. The amino acid sequence of ProQ is shown, divided into NTD, linker and CTD. Peptides measured by mass spectrometry following HDX are shown as solid bars aligned to the corresponding amino acid sequence. The solid bars are sub-divided into four sections from top to bottom to represent the four time points from the experiment (0.3, 3, 30, 300s). The degree of protection from D_2O incorporation into ProQ by the presence of cspE 3'UTR is shown by colour coding according to the bar at the bottom.
