# Supplementary material for: Structure of the Escherichia coli ProQ RNA-binding protein
Source: RNA. 2017 May;23(5):696–711. doi: 10.1261/rna.060343.116 (PMC5393179; doi:10.1261/rna.060343.116)
Supplement: Supplemental Material [file supp_060343.116_Supplemental_Fig_S8.pdf]

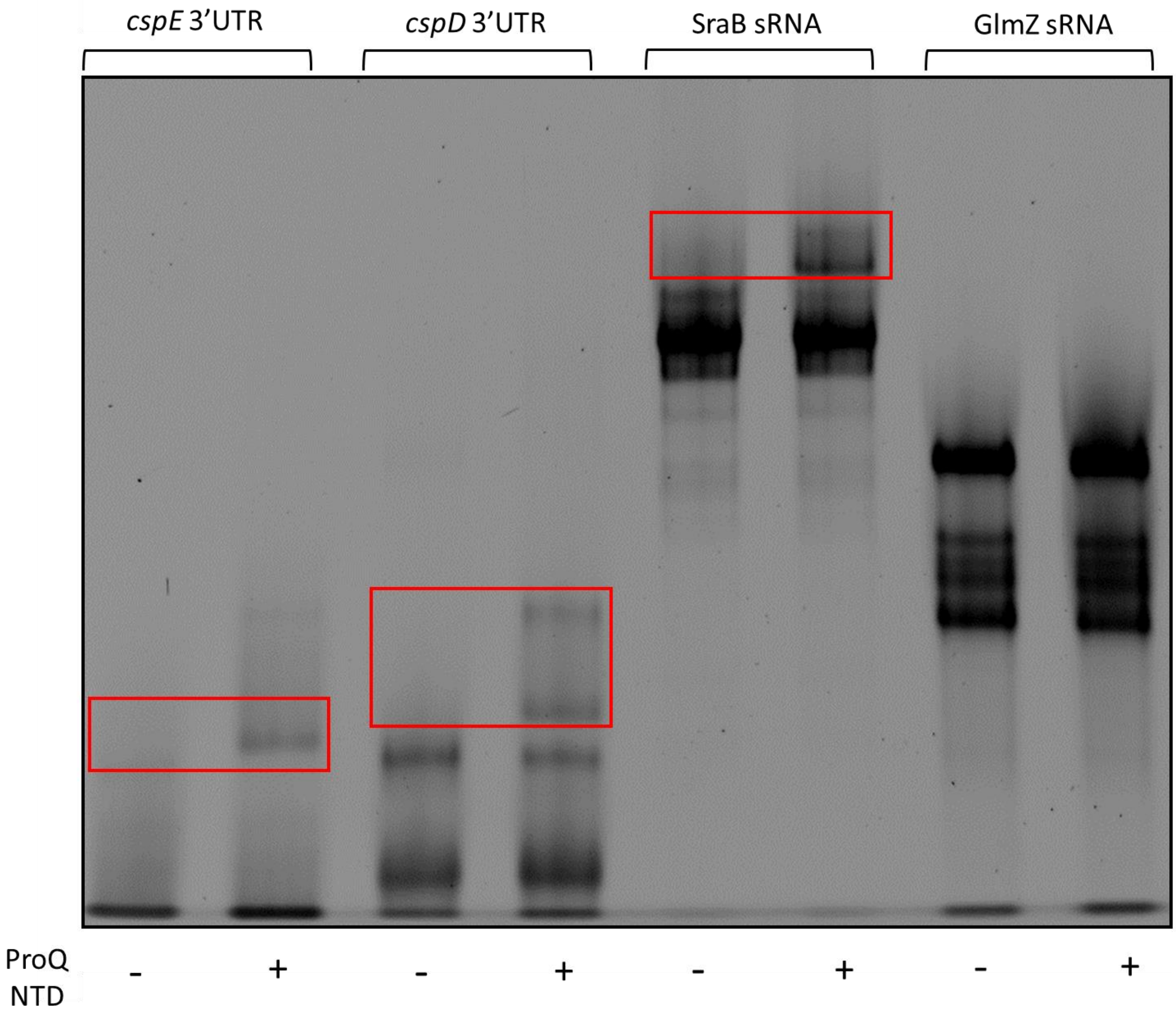

Figure S8. EMSA to show interaction of ProQ NTD with various RNA substrates. The purified ProQ NTD (residues 1-119) was incubated with three known RNA targets of ProQ (cspE 3’UTR, cspD 3’UTR, SraB) and one sRNA that is not considered to be a target of ProQ (GlmZ). Shifted species indicated a complex between ProQ-NTD and RNA are only seen with the known targets of ProQ, and are indicated with red boxes.
