# Supplementary material for: Structure of the Escherichia coli ProQ RNA-binding protein
Source: RNA. 2017 May;23(5):696–711. doi: 10.1261/rna.060343.116 (PMC5393179; doi:10.1261/rna.060343.116)
Supplement: Supplemental Material [file supp_060343.116_Supplemental_Fig_S9.pdf]

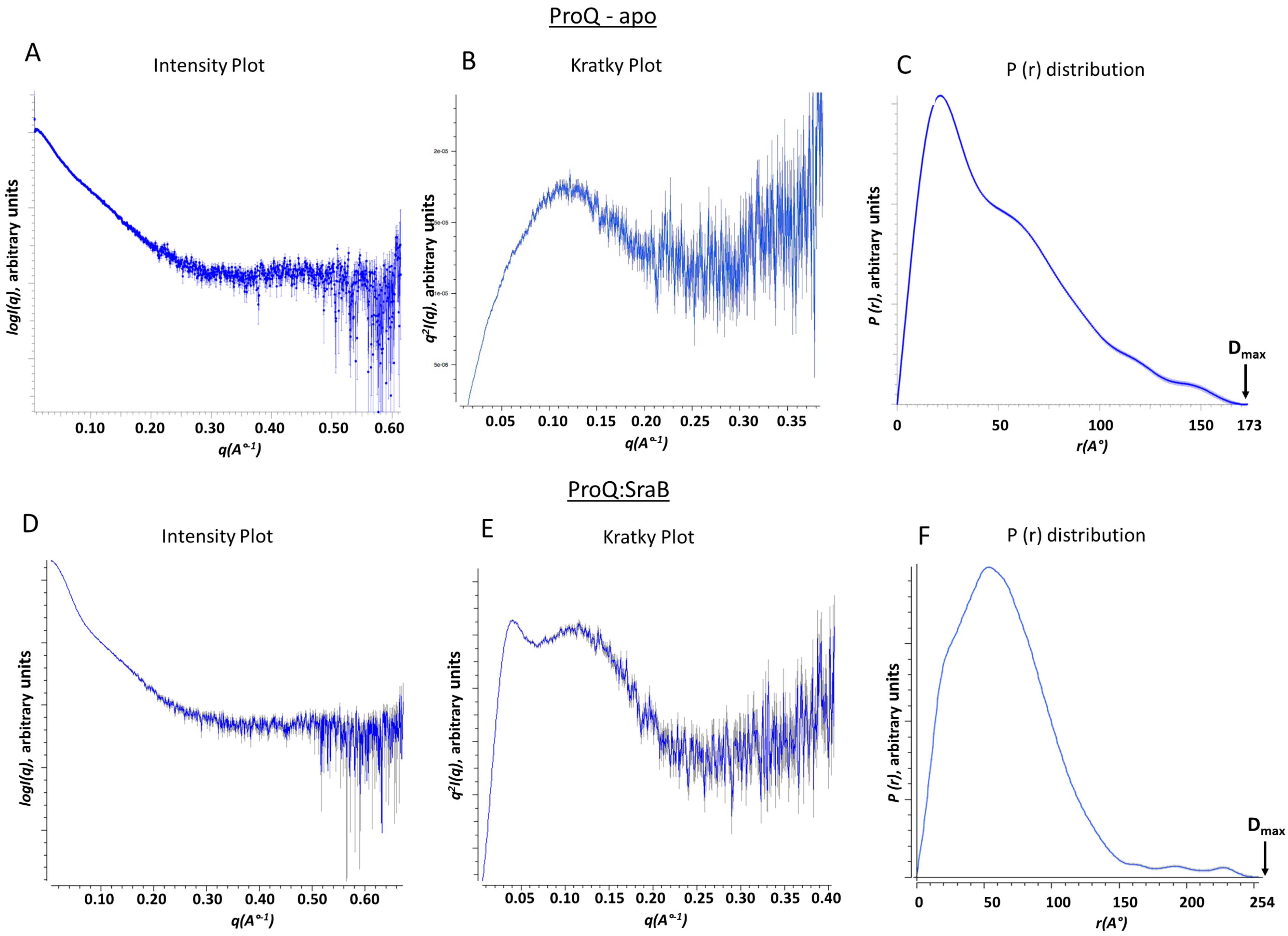

Figure S9. SAXS profiles for ProQ and the ProQ-SraB complex. (A) The scattering profile for ProQ. (B) Kratky plot for ProQ. (C) Distance distribution function P(r) for ProQ. Panels D, E and F show the corresponding profiles for the ProQ-SraB complex.
