# Supplementary material for: Structure of the Escherichia coli ProQ RNA-binding protein
Source: RNA. 2017 May;23(5):696–711. doi: 10.1261/rna.060343.116 (PMC5393179; doi:10.1261/rna.060343.116)
Supplement: Supplemental Material [file supp_060343.116_Supplemental_Fig_S10.pdf]

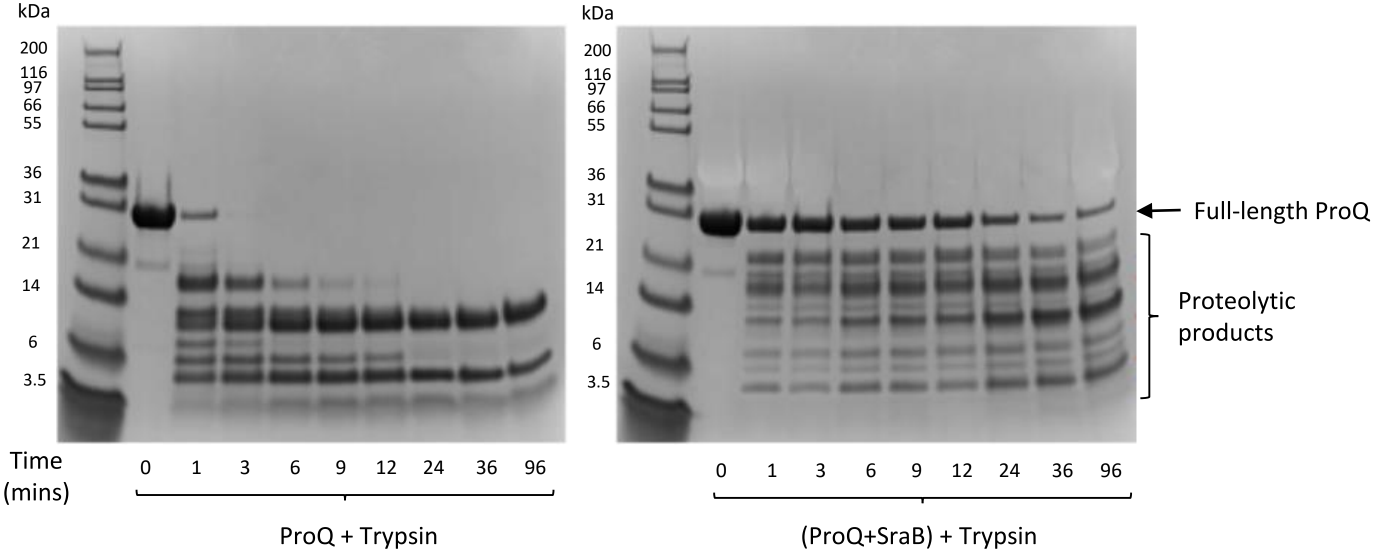

Figure S10. ProQ is protected from trypsin digestion by RNA binding. ProQ (200 µg) was digested with 1 µg trypsin both in the absence (A) and presence (B) if 2-molar excess of RNA (SraB). Coloured arrows denote alternative proteolytic products observed in the absence (red) or presence (blue) of the RNA ligand.
