# Supplementary material for: Structure of the Escherichia coli ProQ RNA-binding protein
Source: RNA. 2017 May;23(5):696–711. doi: 10.1261/rna.060343.116 (PMC5393179; doi:10.1261/rna.060343.116)
Supplement: Supplemental Material [file supp_060343.116_Supplemental_Figure_Legends.docx]

**Figure S1.** SEC-MALS and Analytical ultracentrifugation analysis of ProQ. A) SEC MALS elution profile showing estimated molecular mass variation over the elution profile. B) SDS-PAGE analysis of the peak from the SEC-MALS profile, demonstrating the purity of the sample. C) Summary of molecular mass estimates (Mn – number averaged, Mw- weight averaged) and polydispersity. D) AUC analyses - the velocity sedimentation profile and residuals are shown in the upper panels, and distribution plots as Svedberg units and Daltons are shown in the lower panels.

**Figure S2. NMR data for full length ProQ.** [^1^H,^15^N]-HSQC spectrum of full length ProQ, showing residue assignments for backbone amide sites. Pairs of resonances from side-chain amide sites are connected using magenta lines. Signals from the NTD are indicated in blue, from the linker domain in yellow and the CTD in green.

**Figure S3. NMR data for ProQ-NTD.** (Top) [^1^H,^15^N]-HSQC spectrum of ProQ-NTD, showing residue assignments for backbone amide sites. Pairs of resonances from side-chain amide sites are connected using magenta lines. (Bottom) Assignments for the closely spaced signals contained in the box shown in the upper panel.

**Figure S4. Histogram showing average HN chemical shift differences between the full length and NTD samples of ProQ plotted as a function of residue number.** Average shift differences were determined using the equation Δδ_HN_ = √( Δδ_H_^2^ + 0.15×Δδ_N_^2^ ).

**Figure S5. ProQ binds full length and truncated target RNA ligands at a 1:1 ratio.** SEC-MALS analysis of ProQ with sRNAs derived from the 3’UTR of *cspD* (top) and *cspE* (bottom). Left panels - SEC MALS elution profile showing estimated molecular mass variation over the elution profile. Middle panels - SDS-PAGE analysis of the peak from the SEC-MALS profile, demonstrating the purity of the sample. Right panels - Summary of molecular mass estimates (Mn – number averaged, Mw- weight averaged) and polydispersity.

**Figure S6. Peptide coverage for ProQ:SraB HDX experiment.** The amino acid sequence of ProQ is shown, divided into NTD, linker and CTD. Peptides measured by mass spectrometry following HDX are shown as solid bars aligned to the corresponding amino acid sequence. The solid bars are sub-divided into four sections from top to bottom to represent the four time points from the experiment (0.3, 3, 30, 300s). The degree of protection from D_2_O incorporation into ProQ by the presence of SraB is shown by colour coding according to the bar at the bottom.

**Figure S7. Peptide coverage for ProQ:cspE 3’UTR HDX experiment.**  The amino acid sequence of ProQ is shown, divided into NTD, linker and CTD. Peptides measured by mass spectrometry following HDX are shown as solid bars aligned to the corresponding amino acid sequence. The solid bars are sub-divided into four sections from top to bottom to represent the four time points from the experiment (0.3, 3, 30, 300s). The degree of protection from D_2_O incorporation into ProQ by the presence of cspE 3’UTR is shown by colour coding according to the bar at the bottom.

**Figure S8. EMSA to show interaction of ProQ NTD with various RNA substrates.** The purified ProQ NTD (residues 1-119) was incubated with three known RNA targets of ProQ (cspE 3’UTR, cspD 3’UTR, SraB) and one sRNA that is not considered to be a target of ProQ (GlmZ). Shifted species indicated a complex between ProQ-NTD and RNA are only seen with the known targets of ProQ, and are indicated with red boxes.

**Figure S9. SAXS profiles for ProQ and the ProQ-SraB complex.**

1. The scattering profile for ProQ. (B) Kratky plot for ProQ. (C) Distance distribution function P(r) for ProQ. Panels D, E and F show the corresponding profiles for the ProQ-SraB complex.

**Figure S10. ProQ is protected from trypsin digestion by RNA binding.** ProQ (200 μg) was digested with 1 μg trypsin both in the absence (A) and presence (B) if 2-molar excess of RNA (SraB). Coloured arrows denote alternative proteolytic products observed in the absence (red) or presence (blue) of the RNA ligand.
