# Supplementary material for: Structure of the Escherichia coli ProQ RNA-binding protein
Source: RNA. 2017 May;23(5):696–711. doi: 10.1261/rna.060343.116 (PMC5393179; doi:10.1261/rna.060343.116)
Supplement: Supplemental Material [file supp_060343.116_Supplemental_Fig_S2.pdf]

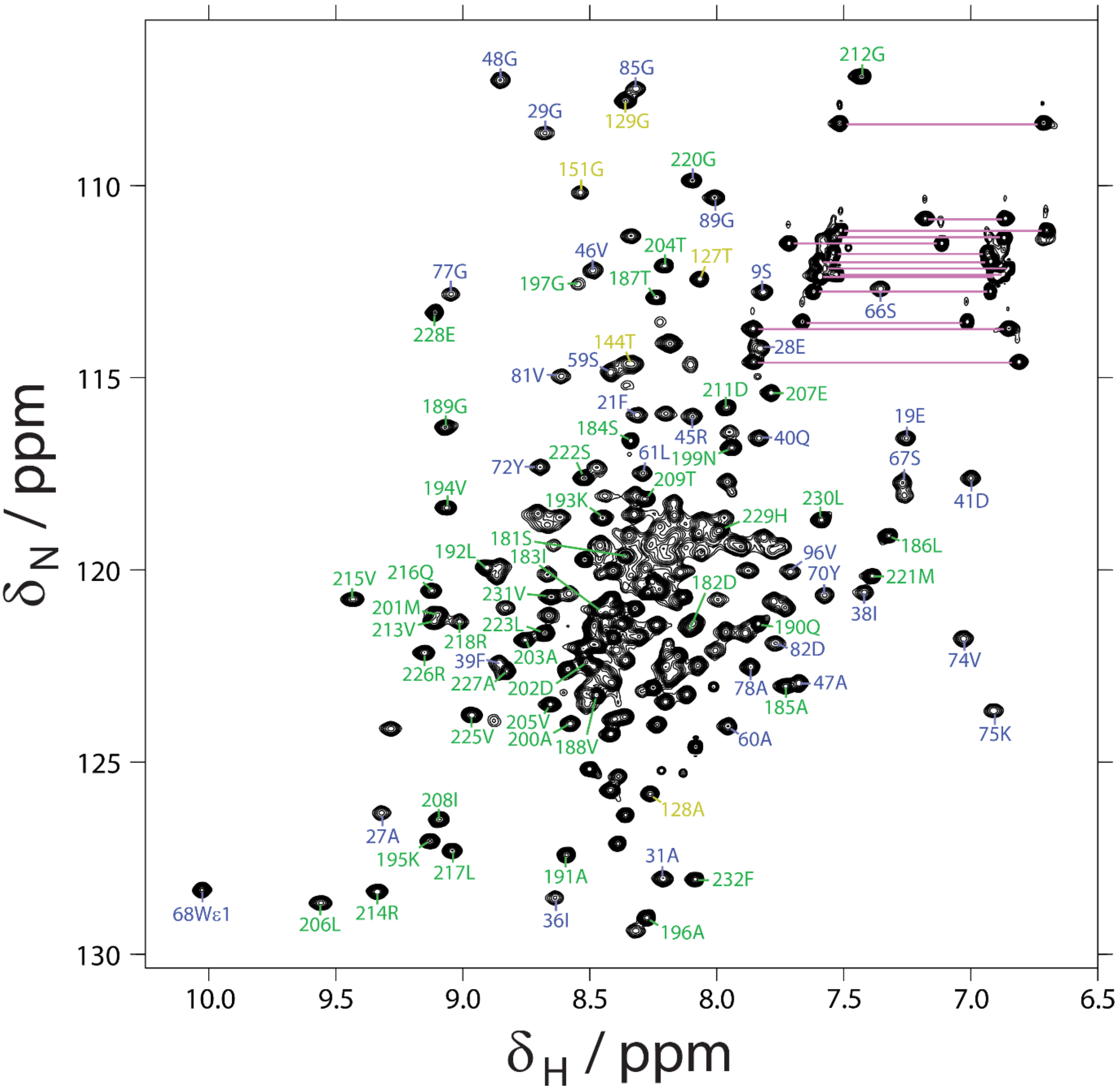

Figure S2. NMR data for full length ProQ. [^{1}H,^{15}N]-HSQC spectrum of full length ProQ, showing residue assignments for backbone amide sites. Pairs of resonances from side-chain amide sites are connected using magenta lines. Signals from the NTD are indicated in blue, from the linker domain in yellow and the CTD in green.
